# Gateway to belonging? A mixed-methods study exploring the experiences of widening participation students in a UK medical school

**DOI:** 10.1136/bmjopen-2026-123003

**Published:** 2026-10-15

**Authors:** Alison Isobel Graham, Katharine Hubbard, Jason William Boland

**Affiliations:** 1 School of Medicine, Newcastle University, Newcastle-upon-Tyne, UK; 2 Teaching Excellence Academy, Buckinghamshire New University, High Wycombe, UK; 3 Wolfson Palliative Care Research Centre, Hull York Medical School, University of Hull, Hull, UK; 4 Health Professions Education Unit, Hull York Medical School, University of York, York, UK

**Keywords:** Education, Medical, Health Education, MEDICAL EDUCATION & TRAINING, Schools, Medical

## Abstract

**Abstract:**

**Objectives:**

To contrast the perceptions of Gateway-entry and Year 1 direct-entry students on their medical school experience when in Year 2 of the medical degree programme.

**Design:**

A two-phase sequential mixed-methods study consisting of a voluntary, anonymous online questionnaire with closed and open questions, followed by nested qualitative semi-structured interviews. Non-parametric Mann-Whitney U tests were used for statistical comparisons. Free-text comments from the questionnaire and interview transcripts were analysed using qualitative content analysis to gain deeper insight into student perceptions.

**Setting:**

A medium-sized UK medical school offering two routes of entry: direct entry into Year one or entry into the Gateway programme.

**Participants:**

Sixty-nine Year 2 MBBS students (15 who entered through the Gateway route and 54 who entered Year 1 directly) responded to a questionnaire. Six participants (five direct-entry, one Gateway-entry) were interviewed.

**Results:**

The questionnaire response rate was 29% (69/242). While Gateway-Yr2 respondents were more likely to report feeling disadvantaged (p=0.039), they were also more likely to feel prepared academically than direct-entry respondents (p=0.047) and they reported similar levels of non-academic preparedness, sense of belonging and support. Gateway-Yr2 were more likely to undertake paid work, but, despite this, 80% of Gateway-Yr2 respondents reported having the work-life balance they would like (compared with 52% of direct-entry respondents).

**Conclusions:**

Widening participation initiatives increase diversity within medical schools, but students entering through these routes often face challenges. To our knowledge, this is the first mixed-methods study to explore the relationship between entry route to medical school and aspects of student experience known to underpin student integration and retention. Findings suggest Gateway programmes may support student integration and academic confidence, helping to bridge disparities in background and enhance institutional familiarity.

**STRENGTHS AND LIMITATIONS OF THIS STUDY**

- Multiple dimensions of the student experience linked to institutional integration and retention were examined.
- The mixed-methods design enabled triangulation of quantitative and qualitative findings, increasing interpretive depth.
- All participants were in Year 2 of the MBBS programme, allowing comparison at a similar stage of training.
- Evaluating at Year 2 offers key early insights but limits conclusions about longer-term impacts.
- The study was conducted at a single institution with a relatively small sample size, which may limit generalisability.

## Introduction

 Patients want, and have better rapport with, doctors who are from a similar background.^1–4^ There is a UK-wide drive to recruit more medical students from widening participation (WP) backgrounds.^5^ Broadly, WP encompasses students from traditionally low-participation backgrounds in higher education.^5^ In the context of medical education, this includes students from lower socio-economic backgrounds, students who have had fewer educational opportunities and students with less access to professional networks than traditional medical students.^6–8^

WP initiatives, such as Gateway/Foundation programmes, have increased medical student diversity.^5^ In the UK, the primary route into undergraduate medicine is direct entry following completion of secondary education at age 18. Traditionally, entry into medical school has been into Year 1 (Yr1) of a 5/6-year programme. Gateway programmes are a route into medical school that offers lower academic entry requirements if specific contextual criteria indicating disadvantage are met.^9^ Gateway students undertake a full academic year of study at university and need to pass exams to enter Yr1 of the standard medical programme. This exposure to university learning, staff and peers, in a smaller group setting than the main medicine programme, allows for an extended transition into independent study.^10^ As well as the formal curriculum (eg, subject-specific knowledge and study skills), Gateway provides an opportunity for students to learn the informal ways of the medical school, get to know staff better and explore how best they learn in a higher education setting.

Although numbers of students with WP characteristics at medical school have increased, it has been found that these students experience difficulties,^11–13^ and those who enter medicine through a Gateway programme have a greater attrition rate and score lower on assessments at graduation.^14^ One risk factor for greater attrition and lower grades is poor student experience.^15^ WP students have also been shown to be more likely to need to undertake paid work, which can impact time available for studying and other activities.^16^ While research on Gateway programmes has largely focused on academic outcomes, progression and attrition,^14 17^ less is known about how students experience medical school after transitioning into the main programme.^18^ In particular, there is limited evidence regarding their perceptions of preparedness, belonging and institutional support despite these factors being associated with student integration and retention.^19^ Understanding these experiences would allow educators to consider how medical schools can best support students entering through Gateway routes.

Student retention is underpinned by academic and social integration into an institution.^19^ Students who do not feel well integrated are more likely to feel isolated and, ultimately, more likely to leave. This is more pronounced for students from disadvantaged or marginalised backgrounds, who are less likely to feel a strong connection to their institution.^19^ Thomas^20^ explains retention through the lens of ‘habitus’, a term coined by Bourdieu to encapsulate the norms and practices of particular social groups, both objective realities and individual understandings of reality.^20–22^ Thomas proposes that if a student feels that their personal habitus and the institutional habitus do not align, then the student is likely to feel like a ‘fish out of water’. They are therefore more likely to return to a more familiar habitus and leave the institution.^20^ An inclusive university will create an institutional habitus that aligns with a wider range of student habitus, thereby increasing the likelihood of feelings of belonging and thus improving retention.^20^ While the overuse of habitus in educational research has been criticised,^23^ it is widely used to explore issues around student belonging and integration into higher education.

This study explores the relationship between entry route into medical school and aspects of the student experience that are known to underpin student integration and retention.^19 20^ It focuses on student self-perception of disadvantage (reflecting academic and social match) and perceptions of academic preparedness, university support structures, employment and work-life balance.^20^ Student perceptions are interpreted as a function of the entry route into university, using the specific context of a Gateway year into medical school. Gateway programmes are designed to widen access to medical school, so take students from a broad range of demographic, socioeconomic, cultural and educational backgrounds.^9^ As such, this study focuses on the relationship between completing Gateway and student perceptions of their sense of integration, which ultimately underpins student retention (figure 1).

**Figure 1:**
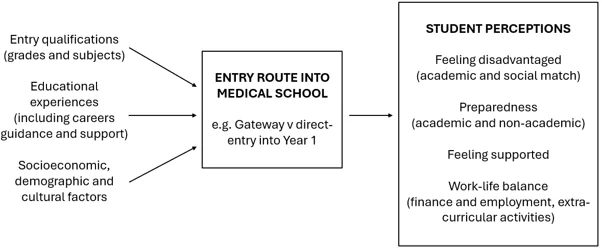
Conceptual framework for this study, building on Bourdieu’s concept of habitus. Student self-perception of disadvantage (reflecting academic and social match) and perceptions of their academic preparedness, university support structures and work-life balance (which includes employment) are interpreted as a function of entry route.

The UK higher education sector is facing numerous challenges. Financial stability is dominating the current discourse^24–26^ but student well-being is also crucial and related. Sense of belonging and connection with their university encourages students to persevere with their course and student life and seek help even through challenging times.^15 20 27^ This helps reduce non-completion rates and produces graduates who are strong ambassadors for the institution, both of which contribute to financial and reputational stability. Consequently, student well-being should be seen not only as supporting individual students and the wider student community but also the stability of the institution itself.

While Gateway programmes are now an established WP route into medicine, evidence regarding their longer-term influence on students’ experiences within medical school remains limited. Through comparison with direct-entry peers, it offers new evidence on the potential ongoing impact of Gateway on aspects of the student experience associated with student integration and retention.

### Aim and research questions

To contrast the perceptions of Gateway-entry and Yr1 direct-entry students on their medical school experience when in Year 2 (Yr2) of the medical degree programme.

Research questions:

- Do students who entered through Gateway have different perceptions of their university experiences compared with direct-entry students?
- Are students who entered through Gateway more likely to undertake paid work and how does paid work impact them?

It was hypothesised that students who entered through Gateway would have increased institutional awareness and thus a sense of belonging due to their longer association with the medical school. Based on previous work, we hypothesise that Gateway students are more likely to need to work^16^ and that they will be impacted negatively by that.

## Methods

A sequential mixed-methods approach, informed by an interpretivist epistemological standpoint,^28^ involving an online questionnaire followed by nested semi-structured one-to-one interviews, was used.^16^ Data were triangulated to increase depth of findings.^29 30^ Qualitative findings are reported alongside the quantitative results to contextualise the quantitative outcomes.

### Educational environment and context

The study took place at a medium-sized medical school in Eastern England offering two routes of entry: direct-entry into Yr1 of the 5-year MBBS programme or entry into the Gateway programme (with reduced academic requirements if specific contextual criteria were met).^31^ The Gateway programme includes classroom-based sessions on theoretical concepts, practical skills and clinical placements. Taught sessions cover core medical sciences (eg, anatomy and pharmacology), clinical and communication skills (eg, physical examination and history-taking), social science (eg, public health and psychology), professionalism, ethics and study skills. The primary focus is facilitating transition from pre-university study to higher education. Following successful completion of the Gateway year, students progress into Yr1, joining the standard medical degree curriculum alongside direct-entry peers.

### Population and sampling

Yr2 MBBS students were chosen as the study population as the first Gateway cohort were in Yr2 at the time of the study. Yr1 direct-entry includes both WP and non-WP students whereas all Gateway students are WP. Respondents in Yr2 who entered through the Gateway programme will be referred to as ‘Gateway-Yr2’. Students in the study cohort who entered through Gateway commenced studies in September 2019 whereas students in this cohort who entered Yr1 directly did so in September 2020 when England was subject to local COVID-19 restrictions (^32^; see online supplemental material 1). The eligible study population comprised 242 students; 220 started the MBBS course directly in Yr1 and 22 entered through Gateway.

### Questionnaire instrument

Questionnaire items were developed following a review of literature relating to belonging, student experiences and support in medical education (eg, ^13 14 33–35^). Question items were developed specifically for this study based on the research aims. Three colleagues (one final-year PhD student and two experienced educators at other medical schools, all with an interest in support of WP students) reviewed the questionnaire items for relevance and clarity. Two current Gateway students reviewed the questionnaire and advertisement text for accessibility, clarity, acceptability and coherence. Minor amendments to phrasing and response options were made in response to their feedback.

The questionnaire contained demographic and closed/open questions on student experiences, administered using a secure online platform (Qualtrics). The questionnaire (online supplemental material 2) consisted of 52–65 questions (up to two questionnaire items/page); estimated 10-min completion time. Adaptive questioning (skip-logic), in which respondents may be directed to different questions based on their previous answers, was used in order to avoid respondents being shown irrelevant questions. There were agreement scale, multiple-choice (both mandatory) and free-text questions (voluntary). Many mandatory questions were followed by a voluntary question encouraging further information. Items were not randomised or alternated so that all respondents were presented with questions in the same order to avoid priming based on previous answers. Respondents could go back to change answers.

Participation was voluntary and anonymous. A link to the questionnaire was emailed to all Yr2 students (n=242) and advertised using posters, in student newsletters and on social media, and mentioned in course news sessions. The survey invitation included details of the study purpose, names of study investigators, details of ethical approval, and the anticipated time for survey completion. Respondents provided electronic informed consent prior to participating. Responses were captured directly in the online survey platform before being downloaded and stored in password-protected cloud storage for analysis. The questionnaire was available for 7 weeks at the start of the autumn term.

Ethical approval was obtained from the Hull York Medical School Ethics Committee (reference 2148, 21 September 2021). The study was carried out in accordance with the Declaration of Helsinki. Informed consent was obtained from all participants. Data were stored confidentially and securely. No incentives were offered.

### Questionnaire analysis

Analysis was performed in RStudio V.1.3.959.^36^ Likert-style questions were numerically re-coded for analysis. ‘Disagree’ was coded as −2 and ‘Agree’ as +2. For the reverse coded question “Have you ever felt disadvantaged?”, ‘No’ was coded as +1, ‘Not sure/other/prefer not to say’ as 0, ‘Yes - occasionally’ as −1 and ‘Yes - many times’ as −2. Cronbach alpha, calculated using the Psych package in RStudio,^37^ deemed the 11 Likert questions presented here to have sufficient internal consistency for analysis (alpha=0.70; appropriate values range between 0.70 and 0.95).^38^ We acknowledge that this Cronbach alpha value is at the lowest end of the appropriate range,^39^ suggesting that the questionnaire items may measure more than one construct, and results should be interpreted with some caution regarding internal consistency.

Analysis of Likert questions used the Likert package.^40^ Statistical comparisons between demographic questions for individual questions used non-parametric Mann-Whitney U tests as some response categories contained small numbers so a normal distribution cannot be assumed.^41^

Participants were classed as WP if they identified with any one of a number of criteria commonly used to define WP status (table 1;^14 34 42 43^). Free-text responses to questionnaire items were analysed using qualitative content analysis.^44^ Responses were read repeatedly to achieve familiarity with the data. Responses were coded according to their meaning, with similar responses grouped into categories developed inductively from the data and categories were refined through iterative review and discussion within the research team. The prevalence of categories was examined to identify commonly reported experiences and perceptions, and illustrative quotations were selected to contextualise the quantitative findings.

**Table 1:** Criteria used to define participants as ‘widening participation’. Definitions of the terms indicated with * are given within the questionnaire found in online supplemental material 2)

For interviews, participants were asked: Do any of the following apply to you? Please respond with simply ‘yes’ or ‘no’. You do not need to say which points apply to you.	For questionnaire respondents, their demographic responses were checked for agreement with any of the categories below.
I undertook the Gateway to Medicine Year. I was the first in my family to go to university. I am from a minority ethnic background. I received free school meals. I am currently, or have previously been, in receipt of an Educational Maintenance Allowance, a 16–19 Bursary, a UCAT bursary or any other bursary. When applying for medical school, I lived in an area of low participation in higher education. (You can check your postcode using the Office for Students website. A postcode being classed as Quintile 1 or 2 using the ‘POLAR4’ measure is usually classed as being in an area of low participation in higher education.) I have a disability or long-term health condition.* I am a carer.* I am a care leaver.* I was over 21 years of age when I started studying. I speak English as an additional language.	

### Interviews

Interest in being interviewed was captured at the end of the questionnaire or by responding to an email. One-to-one interviews with AIG, lasting 35–45 min, were conducted online using Microsoft Teams (Microsoft, Redmond, WA, US) at the start of the spring term. Semi-structured questions, informed by questionnaire results, were used (online supplemental material 3).

Participants were sent a Participant Information Sheet and Consent Form. Interviews were audio-recorded and anonymised before transcription and data analysis. WP participants were identified as described in the previous section.

### Interview analysis

Interview transcripts were read repeatedly to achieve familiarity with the data and analysed using qualitative content analysis.^44^ Responses were coded according to their meaning, with similar responses grouped into categories. Categories were initially grouped inductively in line with the questionnaire topics then deductively for new topics.^45^ Categories were refined through iterative review and discussion within the research team. Interview participants could comment on topic summaries post-analysis (member checking).^46^ No comments were received.

### Data triangulation

Responses to quantitative questionnaire items were analysed first. Data from both the free-text responses to the questionnaire items and interview transcripts were then analysed together to gain deeper insight into the findings from the quantitative items in the questionnaire.

The Standards for Reporting Qualitative Research (SRQR) checklist (^47^; online supplemental material 4) and the standardised Checklist for Reporting Results of Internet E-Surveys (CHERRIES) (^48^; online supplemental material 5) were used when reporting results.

### Researcher characteristics and reflexivity

All members of the research team were experienced tertiary level educators, with JWB also working clinically. JWB was lead for Gateway at the time of data collection and had been the lead at the time Gateway-Yr2 were in Gateway. AIG was part of the medical school but had minimal direct contact with Gateway and Yr2. When conducting interviews, AIG was unfamiliar to participants but was familiar with the educational context. KH was external to the medical school and had no relationship with participants. The researchers recognised that their educational backgrounds and interest in enhancing student learning could influence study design and interpretation and sought colleague and student input on design decisions, for example, questionnaire design. Reflexive discussions within and outside the research team helped challenge assumptions and ensure that findings remained grounded in the data.

## Results

There were 69 questionnaire responses (table 2); response rate 29% (whole cohort=242). The response rate was greater in the Gateway cohort (15/22=68%) than the direct-entry cohort (54/220=25%). Six participants were interviewed (table 3).

**Table 2:** Demographic characteristics of questionnaire participants. Percentages are based on the number of respondents in that category (direct-entry or Gateway). Some categories have been combined for clarity; see online supplemental material 2) for phasing of demographic questions

Category	Demographic	Direct-entry (n=54)	Gateway (n=15)
Gender	Female/woman	31 (57%)	13 (87%)
	Male/man	22 (41%)	2 (13%)
	Non-binary	1 (2%)	0 (0%)
Ethnicity	Asian	20 (37%)	3 (20%)
	Black/other	7 (13%)	0 (0%)
	White	26 (48%)	12 (80%)
Disability	No	47 (87%)	12 (80%)
	Yes	6 (11%)	1 (7%)
	Not sure	1 (2%)	2 (13%)
Free school meals	No	37 (69%)	10 (67%)
	Yes	9 (17%)	4 (27%)
	No response	8 (15%)	1 (7%)

**Table 3:** Demographic characteristics of interview participants

Interviewee designation	Demographic characteristics
A	Female, direct-entry, WP
B	Male, direct-entry, WP
C	Male, direct-entry, WP
D	Female, direct-entry, WP
E	Female, Gateway-entry, WP
F	Male, direct-entry, not WP

WP, widening participation.

### Gateway-Yr2 feel disadvantaged

Gateway-Yr2 respondents were significantly more likely to have felt their background had disadvantaged them during medical school (either occasionally or many times) than direct-entry respondents (Mann-Whitney U=273.5, p=0.039; figure 2).

**Figure 2:**
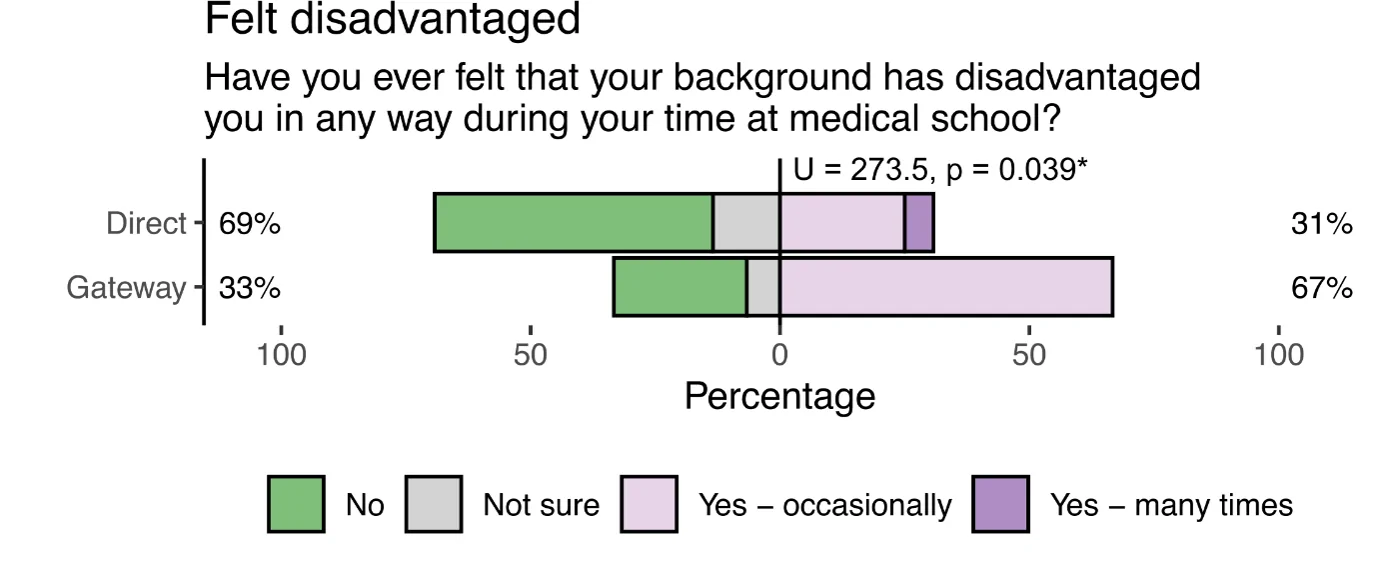
Student perception of disadvantage by entry pathway. Responses of ‘other’ and ‘prefer not to say’ are not presented (n=2). Sample sizes: direct-entry n=54, Gateway n=15. Results of Mann-Whitney U test indicated.

Free-text comments and interviews indicated several explanations behind a feeling of disadvantage (quotes with interviewee letter designations are from interviews; quotes without this are from questionnaire free-text comments). Financial pressures, being first-in-family to attend university and not having family members with a medical background were mentioned together:

When you hear about some people’s experiences due to having family members in the medical field it reminds you where you come from. (Gateway-Yr2 respondent)

The lack of financial stability was compounded by a lack of awareness (among students) of other students’, more precarious, situations:

There are a lot of people that come from more affluent backgrounds; there are some times where people will be quite tone-deaf (laughs) about some of the things they’ll talk about. (Interviewee B)

A lack of family background in medicine or university led to some participants being unaware of informal, tacit information:

Sometimes it seems like other people know more than I do and I’m just like ‘how do you know this?!*’* (Interviewee D)

However, some participants felt that coming from a less advantaged background allowed them to understand patients from deprived backgrounds more easily and be well placed to suggest solutions:

Coming from … a background where there’s a lot more poverty, … it does help in terms of being able to understand other people, … what’s stopping them from being able to access healthcare … once you understand [that] … it’s a lot easier to then be able to come up with solutions as to how to … fix that*.* (Interviewee B)

There was also an appreciation of widening access initiatives:

If it wasn’t for Gateway I wouldn’t even be here in the first place. (Interviewee E)

### Gateway-Yr2 feel more prepared academically

Gateway-Yr2 respondents were significantly more likely to feel prepared academically for Yr2 than direct-entry respondents (U=536, p=0.047; figure 3A). A similar level of significance was not seen with being prepared non-academically (U=440.5, p=0.581; figure 3A).

**Figure 3:**
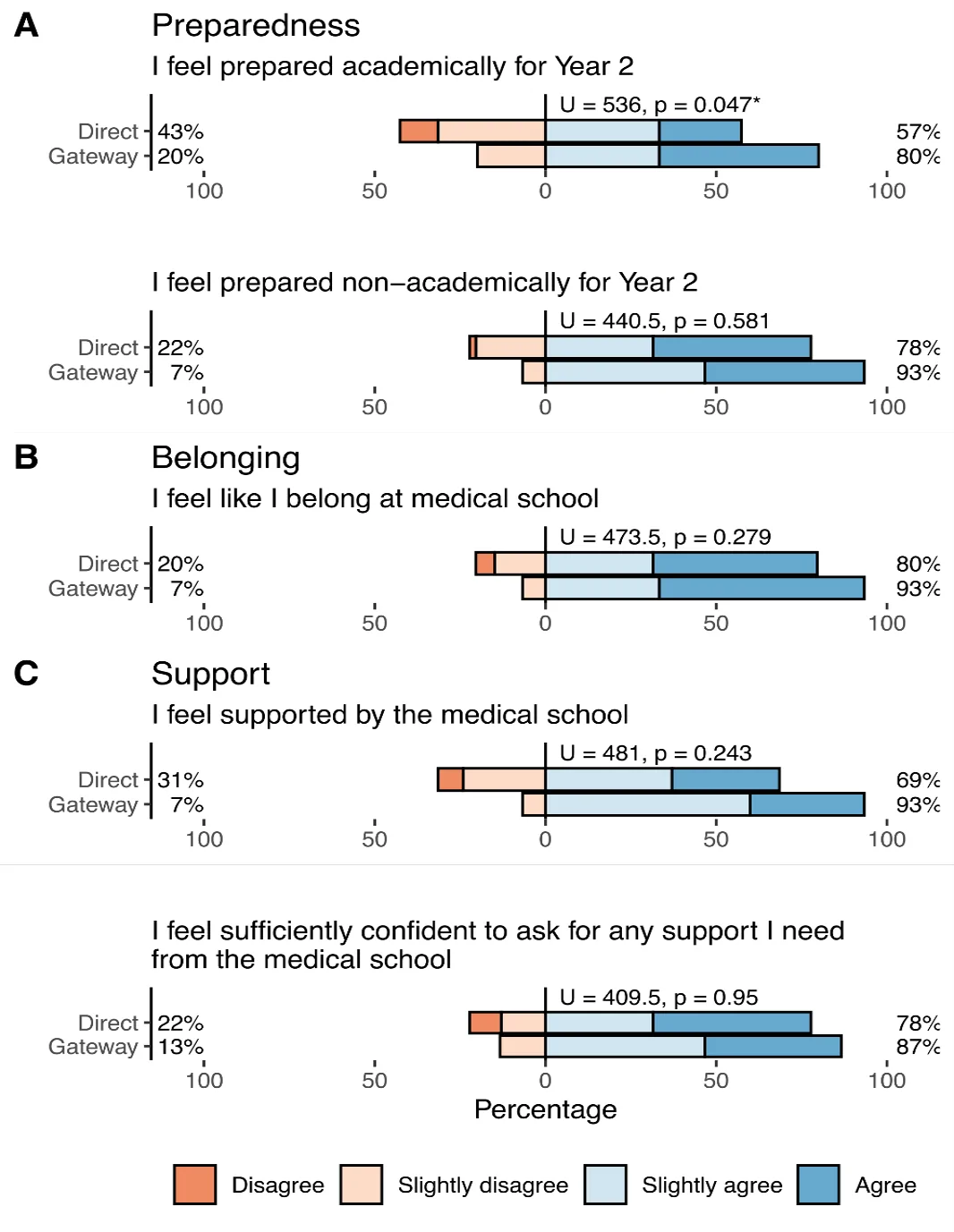
Student experiences of medical school by entry pathway. For each question positive responses (agree) are on the right, negative responses (disagree) on the left. Sample sizes: direct-entry n=54, Gateway n=15. Results of Mann-Whitney U test indicated, significant differences indicated with *.

Reasons participants reported for feeling prepared academically included a supportive environment and revisiting previous content (as part of a spiral curriculum). Several also noted that they felt confident in their communication skills (having had numerous opportunities to practise this in Yr1) but less confident in physical examination skills.

Open-text comments revealed a strong feeling of not ‘being tested’ (specifically in a closed-book setting) and thus feeling less confident in Yr1 material:

I think that I will struggle with exams this year and the pressure of doing them in person when we haven't sat proper exams for over four years. (direct-entry respondent, WP)

The online learning and accompanying isolation experienced by many students during the lockdown restrictions were also mentioned as a reason for feeling underprepared:

It was so difficult being so isolated from other medical students and being online. (direct-entry respondent, WP)

Gateway-Yr2 were asked if they felt Gateway prepared them well for Yr1. All respondents either agreed (93%) or slightly agreed (7%). In free-text comments, participants indicated that this was because of the knowledge, skills and confidence Gateway gave them which they were able to take into Yr1, as well as a recognition that they may not have been able to study medicine had Gateway not been available.

### Belonging

Most respondents reported experiencing a sense of belonging at medical school (57/69=83%), with no significant differences between Gateway and direct-entry cohorts (U=473.5, p=0.279; figure 3B). Free-text responses indicated that key factors supporting a sense of belonging included keeping up with the course workload, working hard and doing well. Factors feeding into a lack of belonging included not feeling smart enough, lacking connection with other students and a perception that participants did not have personality traits aligned with what is expected of medics:

I feel like everyone else is smarter than me. (direct-entry respondent, WP)

### Support

Most participants agreed or slightly agreed that they feel supported by the medical school (51/69=74% agree/slightly agree) and sufficiently confident to ask for support (55/69=80%; figure 3C); however, 14 participants did not feel sufficiently confident to ask for support. No statistically significant differences between Gateway-Yr2 and direct-entry respondents were seen (feel supported U=481, p=0.243; confident to ask U=400.5, p=0.95).

The follow-up free-text question asked if there were any ways the medical school could support students more and encouraged practical examples. Although the question did not name any specific type of support so as not to influence the responses, many responses related to support for academic study, for example, revision sessions, practise question banks, more information about learning outcomes, access to study spaces. For example:

If there were more ways to test your knowledge, because sometimes I feel that I'm not entirely sure if I've learnt something properly or not. (direct-entry respondent, WP)

This echoes what respondents said previously around a lack of feeling of ‘being tested’. A smaller number mentioned financial aid/bursaries. Some respondents also identified current support measures that were useful to them:

I think the medical school does more than enough in terms of supporting my academic and non-academic development. (direct-entry respondent, WP)

### Gateway-Yr2 are more likely to need to undertake paid work

Gateway-Yr2 respondents were significantly more likely to need to undertake paid work (U=249, p=0.012; figure 4A). Eleven out of the 15 (73%) Gateway-Yr2 respondents undertook paid work compared with 19/54 (35%) direct-entry respondents. Within those who undertook any form of paid work (n=30), there were higher proportions of Gateway-Yr2 agreeing that they needed to work to support themselves financially (Gateway 9/11=82%, direct-entry 12/19=63%) and that their paid employment impacts their studies and university experience (Gateway 5/11=45%, direct-entry 6/19=32%), but neither were statistically significant (figure 4B).

**Figure 4:**
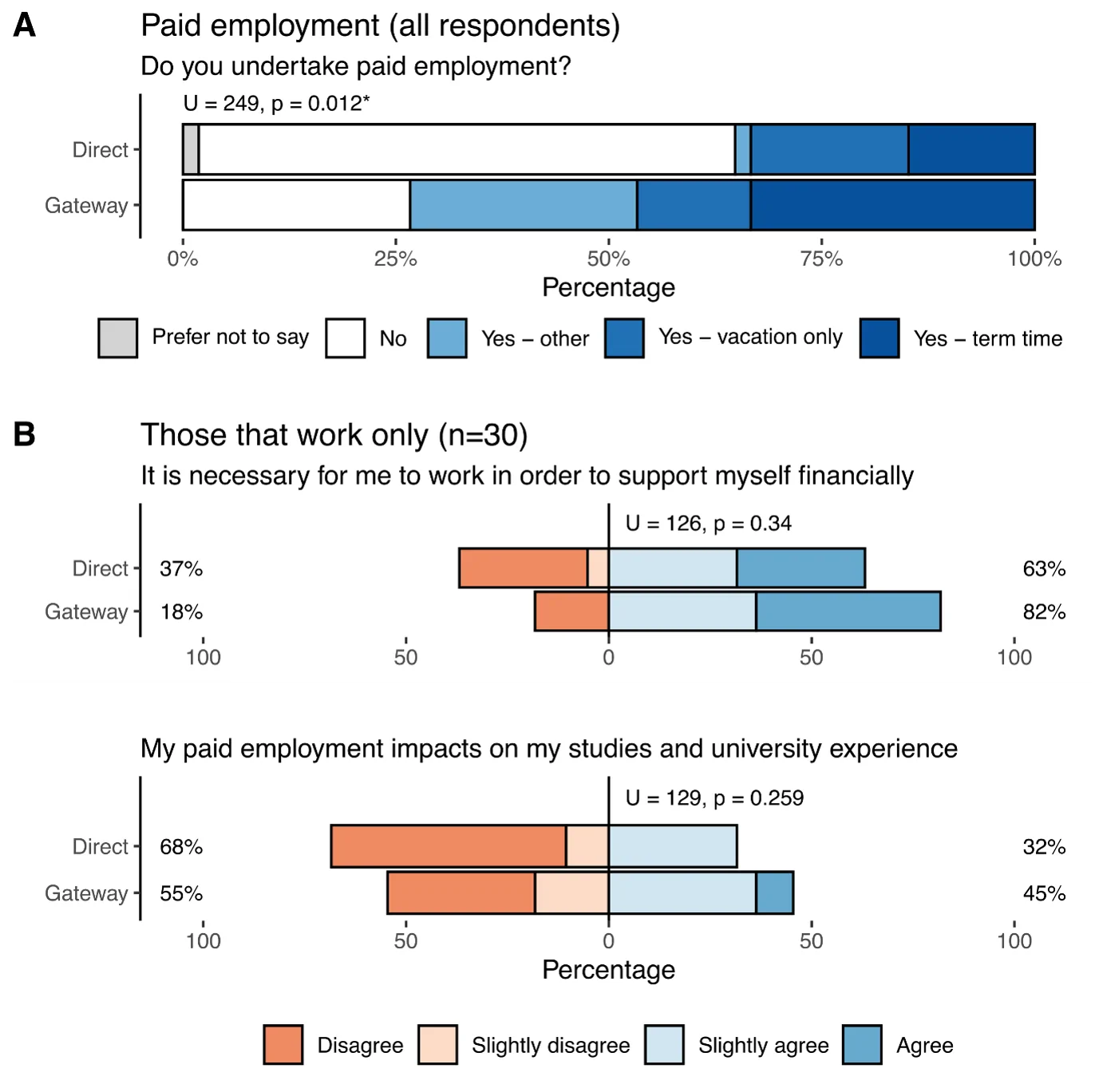
Student paid employment by entry route. Sample sizes: direct-entry n=54, Gateway n=15. Results of Mann-Whitney U test indicated significant differences indicated with *.

Free-text comments revealed that work respondents undertook had many patterns (eg, regular/irregular hours, vacations only, night shifts) and types (eg, student ambassadors, selling home-made items).

Some work was seen as useful for medical studies (eg, vaccine clinic work). Despite this positive aspect for some, the lack of financial security was an issue for others:

I am a bursary student & still struggle financially sososo much*.* (direct-entry respondent, WP)

I also feel that because I have to work to financially support myself through medical school that some people think I don't value my place. (which is not true) (direct-entry respondent, WP)

Some respondents were reluctant to take money from parents because of a lack of spare family money so worked to avoid this.

Needing to work can impact on time available to get experience relevant to the course:

I find that I haven't had time to get as much experience as other people due to having to work and not being able to access such a big variety of healthcare settings. (direct-entry respondent, WP)

### Work-life balance

More Gateway-Yr2 respondents (80%, compared with 52% direct-entry respondents) agreed they had the work-life balance they would like, although this was not statistically significant (U=524.5, p=0.069; figure 5). This was despite Gateway-Yr2 being more likely to work and more likely to have to work to support themselves (figure 4). Free-text comments acknowledged that maintaining an appropriate balance requires effort and planning. Participants were significantly more likely to agree that they *want* to take part in extra-curricular activities than *can* (online supplemental material 6). The main reason was lack of time.

**Figure 5:**
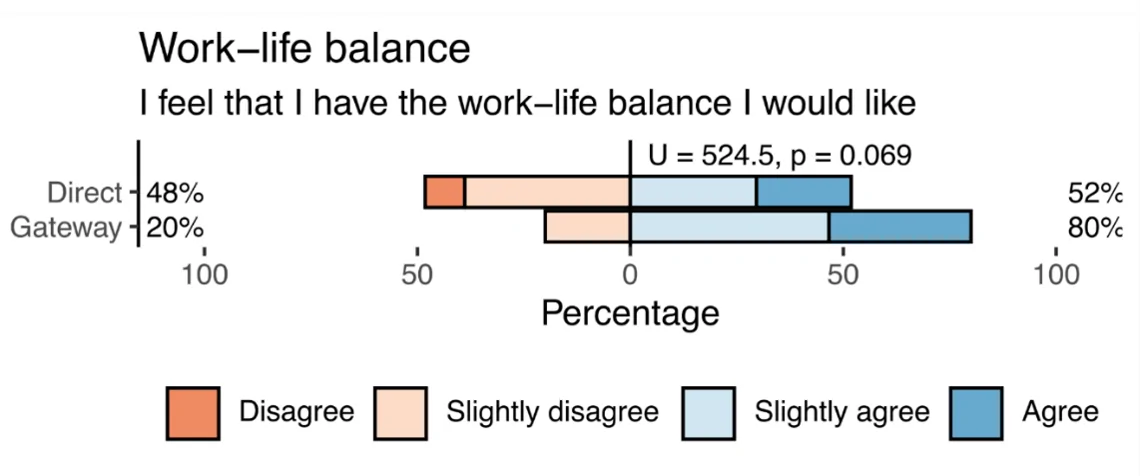
Student perceptions of work-life balance at medical school by entry pathway. For each question positive responses (agree) are on the right, negative responses (disagree) on the left. Sample sizes: direct-entry n=54, Gateway n=15. Results of Mann-Whitney U test indicated.

## Discussion

To our knowledge, this is the first mixed-methods study to explore the relationship between entry route to medical school and aspects of student experience known to underpin student integration and retention. The study aimed to contrast the perceptions of Gateway-entry and Yr1 direct-entry students on their medical school experience when in Yr2 of the medical degree programme. Regarding our first research question, we found that Gateway-Yr2 had different perceptions of their university experiences compared with direct-entry students in some respects but not others: Gateway-Yr2 respondents were significantly more likely to report feeling disadvantaged, but they were also significantly more likely to feel prepared academically than direct-entry respondents, and reported similar levels of non-academic preparedness, sense of belonging and support. Regarding the second research question, Gateway-Yr2 were more likely to undertake paid work but, despite this, most Gateway-Yr2 respondents reported they have the work-life balance they desired. Taken together, we can see that Gateway-Yr2 felt disadvantaged but also felt a sense of belonging; they had to work but also felt they had a reasonable work-life balance. We propose that Gateway may provide opportunities to develop institutional habitus and thus to feel a greater sense of belonging, which is explored below.

### Gateway-Yr2 feel disadvantaged

Gateway-Yr2 respondents were significantly more likely to feel their background had disadvantaged them (figure 2). This question was phrased retrospectively (ie,‘…felt…’) and respondents were free to interpret ‘background’ in their own way. Gateway-Yr2 had an additional year as part of the medical school community at the time of the study so were reflecting on a longer duration than direct-entry respondents. Free-text comments indicated several explanations behind a feeling of disadvantage, including lack of financial resources, financial demands of the course and lack of peer awareness about different financial situations, which echo previous findings from first-in-family students attending a Canadian medical school.^49^ In our study, financial considerations were frequently mentioned at the same time as lacking familial networks as sources of informal information and advice on university life and medicine, which is known to be more challenging for WP students to access.^50^ Some respondents felt there were positives to coming from a less advantaged background as this allowed them to understand the concerns of patients from similar backgrounds; previous research at a Canadian medical school demonstrated similar insights from first-in-family students^51^ and highlights the benefits of diverse medical recruitment to the wider population. Some respondents acknowledged that, without Gateway, they would not have been able to attend medical school.

### Preparedness

Gateway helps students feel prepared for Yr1, feel supported by the medical school and increases sense of belonging (figure 3), which is consistent with previous work.^10^ Gateway-Yr2 respondents were significantly more likely to feel prepared academically for Yr2 than direct-entry respondents (figure 3A), possibly reflecting the situation at the time of the study (ie, Yr1 students began their medical studies subject to restrictions due to the COVID-19 pandemic) and the fact that Gateway is generally taught in smaller classes which is known to impact positively on student satisfaction.^52^ The former point is supported by free-text comments indicating a feeling of low self-confidence in knowledge across the cohort due to a lack of closed-book exams because of the restrictions. Restrictions resulting in the removal of summative exams have been shown to lead to a decreased motivation for learning and fear of gaps in knowledge as well as isolation from peers.^53^

### Belonging and support

Although most respondents felt a sense of belonging (figure 3B), some reasons given for a lack of belonging included not feeling smart enough, lacking connection with peers and a perception of not having the right personality to be a doctor. These all align with the concept of ‘imposter syndrome’ which is well-recognised as being a concern among medical students,^54–56^ and the prevalence of which has even been shown to increase during medical school.^57^ Addressing the underlying causes of imposter syndrome is critical as Pedler *et al*^15^ found that having a sense of belonging increases students’ academic motivation and enjoyment in their studies, which can improve academic achievement, lowering the chance of students considering leaving before completing their studies, thus supporting student retention.

Most respondents also felt supported and confident enough to ask for support (figure 3C). Previous work by O’Sullivan *et al*^35^ with Foundation Year (FY) students showed that the FY increased belonging by providing social networks, long-term relationships and acquisition of ‘navigational capital’, thus increasing alignment with the institutional habitus. Suggestions for additional support from all participants focused on academic support, reflecting the predominance this issue had at the time. Financial concerns were reported but less so, which was unexpected. Although most participants felt confident to ask for support, approximately 20% did not. Further work is required to explore barriers to reaching out.

### Paid work and work-life balance

Gateway-Yr2 were significantly more likely to undertake paid work (figure 4A). It has been shown before that students from lower socioeconomic backgrounds are more likely to need to work.^16^ We hypothesised that Gateway students would be impacted negatively by their increased likelihood of being in paid employment. A higher proportion of Gateway-Yr2 than direct-entry Yr1 students agreed that they needed to work to support themselves financially and that their paid employment impacts their studies and university experience but neither were statistically significant (figure 4B). Additionally, more Gateway-Yr2 respondents agreed they had the work-life balance they would like, although this was also not statistically significant (figure 5). It is not appropriate to draw strong conclusions from these data but this may indicate that Gateway-Yr2 have a more pragmatic view of what is achievable having had more time to establish themselves at medical school; resulting in a more favourable view of their work-life balance. Given that students are more likely to form friendships with students similar to themselves,^13 58^ it is likely that social micro-environments exist. One possible explanation is that Gateway students compare themselves with peers facing similar (economic) constraints. O’Sullivan *et al*^35^ showed that FY students saw other FY students as ‘their group’ which helped with belonging whereas direct-entry WP students reported higher levels of disconnection and isolation due to having a different comparator group that they found ‘hard to break into’. As paid work impacts academic achievement^33^ and friendship groups are related to subsequent exam performance,^58^ both have the potential to impact the attainment gap between WP and non-WP students.^59^

### Strengths and limitations

The study explored multiple dimensions of student experience linked to institutional integration and retention, including belonging, preparedness, support and paid work. The mixed methods design enabled triangulation of quantitative and qualitative findings, increasing interpretive depth. All participants were in Yr2 of the MBBS programme, allowing comparison at a similar stage of training, although Gateway-Yr2 students had completed an additional year at university. This temporal difference between cohorts may have influenced preparedness, peer relationships, institutional familiarity and sense of belonging independently of entry route.

We chose to study Yr2 students as a single timepoint of particular interest; it includes transition periods (to university and, for some, from Gateway to Yr1) and is the period when many foundational educational and social experiences are made. Practically, the first cohort of Gateway students at this institution were in Yr2 at the time of the study. Insights from this timepoint allow consideration of early interventions. We acknowledge this is early in medical training and future longitudinal work is required to determine whether these observations persist throughout medical school and into postgraduate training.

Gateway-Yr2 experienced approximately 4 months of standard university life before the COVID-19 lockdown restrictions began in England. In comparison, the students who entered Yr1 directly began university when local COVID-19 restrictions prevented in-person teaching taking place (see online supplemental material 1 for more information). These restrictions are very likely to have magnified issues and reduced contentment in the study population,^60 61^ particularly in students from WP backgrounds.^62^

Gateway entrants are a less heterogeneous group than Yr1 direct-entry students, as all Gateway students met WP criteria whereas the Yr1 direct-entry cohort included both WP and non-WP students. The study was conducted at a single institution with a relatively small sample size and self-selecting participants which may limit generalisability.

### Future work

The small sample size showed patterns in the data but these should be confirmed with a larger, targeted study, undertaken post-COVID-19 restrictions. Longitudinal follow-up of this cohort, and Gateway students at other institutions, will enable greater understanding of the continued impact of Gateway, and comparisons with other groups. Recent research has shown that graduates from Gateway programmes face disadvantage when applying for specialty training,^63^ highlighting the importance of longer-term follow-up.

There are wider influences on belonging which should be borne in mind in any future investigations. This includes larger cohort sizes and online learning becoming more common, which can reduce the availability of personal learning opportunities in both clinical and non-clinical settings.

We hypothesise that concerns about finances will increase in this cohort as they move through the course and in future cohorts. With more students undertaking paid work and the cost-of-living crisis,^64 65^ learning more about the quantity and type of work medical students undertake, and its impact, would be useful, especially as WP numbers increase. This will enable characterisation of strategies to support WP students through medical school to become resilient doctors.

### Implications

This study explored the relationship between entry route and aspects of student experience known to underpin student integration and retention.^19 20 66^ Gateway-Yr2 were more likely to report feeling disadvantaged, but this perceived disadvantage is not evident in responses to other questions. If there is a gap between perceptions and reality, how much does one influence the other? By definition, Gateway students are from disadvantaged backgrounds and have frequently overcome barriers to be at university.^49 67–69^ While they may feel generally/historically disadvantaged, specific areas, such as belonging, preparedness and support, were unaffected. Students from disadvantaged backgrounds are less likely to feel institutional belonging^19^ but this study showed this was not the case for Gateway-Yr2 respondents.

Gateway-Yr2 were more likely to report that they feel prepared academically which poses the question of whether any practices/activities in Gateway could be useful for Yr1 students and specifically WP students who enter Yr1 directly. Throughout their time at university, direct-entry WP students would benefit from support that goes beyond the academic^35^ to increase their familiarity with the institutional habitus. Direct-entry WP students may also benefit from peer support from others they see as being from similar backgrounds as them.^56^ Medical school and university represent landscapes of tacit knowledge, with expectations and social norms, that can be unknown to those outside these environments. In the context of a FY for international students, Jones *et al*^70^ discuss how peer support networks and strong working relationships with staff are key in building student belonging, increasing confidence in asking for help, and helping to develop institutional habitus. A Gateway Year can be likened to an International FY in that both prepare groups of students to function in a space that is unfamiliar to them.

Knowing that Gateway students are more likely to undertake paid work, there is the opportunity for staff and central services (eg, careers) to signpost students to work that is less impactful on their studies (eg, student ambassadors) or supports their medical development (eg, healthcare assistant^71^). Providing more support for current WP students and ensuring students can financially support themselves have both been highlighted as priorities for the next 10 years.^6^

## Conclusion

By Yr2 of the medicine programme, students who entered through a Gateway programme were significantly more likely to report feeling disadvantaged, despite being significantly more likely to feel prepared academically and feeling a sense of belonging and support. They are significantly more likely to undertake paid work but despite this, most reported having their desired work-life balance. The findings suggest the impact of Gateway may still have been evident in Yr2 through enhanced familiarity with the institutional habitus.

Our findings suggest Gateway may provide protective factors that support integration and potentially retention, though financial barriers persist. Gateway courses provide intentional, organised support for academic, clinical and professional readiness that helps students transition into the demands of medical school, which are likely to benefit all students but particularly those from WP backgrounds. Medical schools should consider providing Gateway-like practices for direct-entry WP students to enhance inclusion and mitigate inequities. Further research with larger, longitudinal cohorts is warranted to clarify the impact of preparedness, belonging and paid work on attainment and attrition. Supporting WP students holistically is not only a matter of equity but also essential for sustaining a diverse, resilient medical workforce.

## Supplementary material

10.1136/bmjopen-2026-123003 online supplemental file 1

## Data Availability

Data are available upon reasonable request.
