# Supplementary material for: Gateway to belonging? A mixed-methods study exploring the experiences of widening participation students in a UK medical school
Source: BMJ Open. 2026 Oct 15;16(10):e123003. doi: 10.1136/bmjopen-2026-123003 (PMC13691842; doi:10.1136/bmjopen-2026-123003)
Supplement: Supplementary file 1 [file bmjopen-2026-123003.reviewer_comments.pdf]

PEER REVIEW HISTORY

BMJ Open publishes all reviews undertaken for accepted manuscripts. Reviewers are asked to complete a checklist review form and are provided with free text boxes to elaborate on their assessment. These free text comments are reproduced below.

ARTICLE DETAILS

Title (Provisional)

Gateway to Belonging? A mixed-methods study exploring the experiences of widening participation students in a UK medical school

Authors

Graham, Alison Isobel; Hubbard, Katharine; Boland, Jason

VERSION 1 - REVIEW

Reviewer 1

Name Nikbakht Nasrabadi, Alireza

Affiliation Tehran University of Medical Sciences, School of Nursing and Midwifery , Medical-Surgical Nursing

Date 14-Jun-2026

COI none

Informative useful study

Reviewer 2

Name Keshmiri, Fatemeh

Affiliation Shahid Sadoughi University of Medical Sciences and Health Services, Medical Education Department

Date 24-Jun-2026

COI none

I would like to thank you for providing the opportunity to review this paper.

The article addresses an important topic, and the suggestions made could be helpful.

Abstract

The methods section in the abstract is too brief and should be written in more detail.

Introduction

The novelty of the work and the research gap need to be given more attention by the authors.

The introduction should be rewritten according to the journal's style. Some parts toward the end of the introduction also require serious revision in line with the journal's formatting guidelines.

Methods

The study population and sample size need to be clearly described.

In the setting section, more information should be provided regarding the educational program implemented in the target population.

The process of quantitative data collection requires further explanation.

The qualitative data analysis process is unclear; the approach, analysis procedure, and steps need to be elaborated.

The method of integrating quantitative and qualitative data is not specified.

Results

The study findings need to be rewritten in accordance with the study objectives.

**Reviewer**	**3**
**Name**	**Hu, Zijing**
**Affiliation**	**University of Johannesburg, Complementary Medicine**
**Date**	**01-Jul-2026**
**COI**	**none**

Clarify the questionnaire development and validation process in slightly more detail.

**Reviewer**	**4**
**Name**	**Johnston, Peter**
**Affiliation**	**NHS Education for Scotland, North Deanery, Pathology**
**Date**	**21-Jul-2026**
**COI**	**none**

Overall evaluation and general comments

This is an interesting and well researched study that looks at widening participation in a single medical school in England. It is set in COVID – my assumption is that, if this is like other work, COVID will have magnified issues and reduced contentment in the study population. The study provides insights into the perceptions of students of gateway compared to others in the programme some of whom are also from similar backgrounds to the gateway group and others from more diverse origins. The study suggests gateway eases the route to university/medical programme satisfaction however the authors do accept they are looking at one group who have two years and another with mostly one year (although including students with previous degree) experience.

Learning for me is in the way the gateway programme seems to make the move to the university process better tolerated which is a positive finding, although it is tempered by the fact the study stops at year two when most students will have another 3 years before completion and several years in residency programmes thereafter before reaching career destinations.

Title and abstract

The title reflects the content of the paper and catches the eye. The abstract accurately reflects the content.

Introduction and conceptual framework

The introduction sets the scene well. I have no real issues. I do wonder when it became "traditional" for medical students to be from well-heeled, university educated backgrounds? There was a time (in living memory – mine, for example!) when most of the class were students whose parents had been in or had to deal with the aftermath of WW2, rationing, conscription and the like. I've never seen a time frame set of the evolution to what we now see. I guess this is me musing but I do wonder about the term "traditional".

As regard the conceptual framework, the authors are quite clear in how they set up the study, its structure and theoretical models. I do wonder if when looking at belonging, student number has a role to play? As class sizes mushroom, the ability of staff to provide personal learning opportunities declines, made worse by the apparent attitude on NHS employers in job planning, reducing the time and threatening the ethos of education in the NHS. These may be confounding factors, as may the move in COVID and beyond to more and more online delivery of courses. It might be useful if the authors referenced these other unhelpful pressures in the context of belonging, particularly as they do include a section on wider context which touches on these points.

Methodological rigour

Questionnaire instrument

I have no issue with the Likert scales. Looking at the question on “words that describe how you feel” (P52, l30) I was struck by the negativity of the words listed. This may well reflect reality but I felt 13 of the 37 were positive whilst the remaining 24 embraced negative perceptions. If someone were to complete this at random, then the likelihood of being negative nearly 3:1 (approx.). I possibly would have just left blank boxes and asked for up to three words positive and three negative to see what we got rather than making the suggestions. I think the authors should comment on this point. (And reference the instrument in the text as appearing in the supplementary material.)

Questionnaire analysis

I feel the Cronbach’s alpha (P12 l46-51) of 0.70 is on the borderline of acceptability – this is stated as the range of “appropriate values” is quoted. A comment on this might be useful to help establish how the authors dealt with this figure given these cut offs have justifications. The use of non-parametric methods is agreed as correct.

Table 1 (P14) mentions the term “care leaver”. I assume this means someone who was in care at some point but it might be helpful to state that in brackets after the term to ensure clarity.

Interview questions

Supplementary material 3 (P59-60) has the question set. A specific question is there about feelings of preparedness for gateway students but there is no easy way to see how this topic was introduced to direct entry students. Would the authors please explain how they ensured that this topic was broached in the interviews. This point does not detract from the information gained in the interviews, but I suspect it does potentially impact on the way the authors compare the groups, even in qualitative work.

Results

Results are clearly presented but note the point about comparisons above.

Discussion and conclusions

The conclusions are clearly stated. The authors do not overstate the significance of the findings rather they are open to the need for further exploration. How feasible it is to tease out factors in a complex and dynamic environment where people are continually developing their professional identity is an interesting point.

The strengths and limitations (P27 l54-P28 l28) and the summary (P4) seem to me to stress what was done rather than the relevance and possible outcomes. I think a limitation is the timing of the study at year 2 which may be quite early to see if there is long-term benefit. Of course, the longer you wait the more difficult it is to sort out cause and effect that discussion is, I think, a relevant point and it might be helpful to explain why the study was done when it was done. UKMED data research is tending to show a change in residency outcomes to a wider list of specialty career choices in WP groups than the first such study and this might

usefully be referenced here. Finally, I am pretty sure COVID will have been influential in this study – but that does not upset the findings, just how the story is told around these, given we mostly felt quite adrift in a sea of uncertainty in the pandemic.

Clarity length and ethical approval

The paper is seriously and quite meticulously written. I cannot fault that. The authors might think about the way the story unfolds and how this lands – although I concede this is a point of style over substance.

Ethics – no issue.

EDI

Societal structure and function and the inequalities these surface are the essence of the paper.

VERSION 1 - AUTHOR RESPONSE

**Reviewer 1**		
Informative useful study	We thank Reviewer 1 for their positive assessment and appreciate these encouraging comments.	
**Reviewer 2**		
I would like to thank you for providing the opportunity to review this paper.<br>The article addresses an important topic, and the suggestions made could be helpful.	We thank Reviewer 2 for their detailed and constructive review.	
Abstract<br>The methods section in the abstract is too brief and should be written in more detail.	The detail about the methods in the abstract has been expanded to provide additional detail regarding study design, participants, and analysis methods while remaining within the abstract word limit.	The Design bullet point now reads:<br>“Design: A two-phase sequential mixed-methods study consisting of a voluntary, anonymous online questionnaire, with closed and open questions, followed by nested qualitative semi-structured interviews. Non-parametric Mann-

		Whitney U tests were used for statistical comparisons. Free-text comments from the questionnaire and interview transcripts were analysed using qualitative content analysis to gain deeper insight into student perceptions."<br><br>The Participants bullet point has been amended to provide more detail about the six participants that were interviewed: "Six participants (five direct-entry, one Gateway-entry) were interviewed".<br><br>The following statement has been added to the Results bullet point: "The questionnaire response rate was 29% (69/242)."
Introduction<br>The novelty of the work and the research gap need to be given more attention by the authors.	We have revised the Introduction to better articulate the current evidence base, identify the existing knowledge gap, and clarify how our approach contributes novel insights.	The sentence addressing the research gap has been expanded to: "Whilst research on Gateway programmes has largely focused on academic outcomes, progression and attrition [14,17], less is known about how students experience medical school after transitioning into the main programme [18]. In particular, there is limited evidence regarding their perceptions of preparedness, belonging

		and institutional support despite these factors being associated with student integration and retention [19]. Understanding these experiences would allow educators to consider how medical schools can best support students entering through Gateway routes.”<br><br>A new paragraph has been added to address novelty:<br>“While Gateway programmes are now an established widening participation route into medicine, evidence regarding their longer-term influence on students' experiences within medical school remains limited. Through comparison with direct-entry peers, it offers new evidence on the potential ongoing impact of Gateway on aspects of the student experience associated with student integration and retention.”
The introduction should be rewritten according to the journal's style. Some parts toward the end of the introduction also require serious revision in line with the journal's formatting guidelines.	We are unclear exactly how the Introduction is not in line with the formatting guidelines but think it might relate to our use of sub-headings so these have been removed.	Sub-headings have been removed from the Introduction. We have retained a section called “Aim and research questions”. The statement about epistemological standpoint has been moved to the Methods.

	The Introduction has been reviewed and revised to improve flow.<br><br>If there are any further revisions required to align with the journal's formatting guidelines, we would welcome the opportunity to do those.	The Introduction has been re-ordered to improve flow.<br><br>Other minor amendments to improve flow are noted in tracked changes.
Methods<br>The study population and sample size need to be clearly described.	Additional detail has been added describing participant eligibility, recruitment, sample characteristics and response rates.	The study population is already described in the "Population and sampling" section. The following sentence has been added to provide additional information: "The eligible study population comprised 242 students; 220 started the MBBS course directly in Yr1 and 22 entered through Gateway."<br><br>Information about recruitment is provided in the "Questionnaire instrument" section and response rates are in the Results.
In the setting section, more information should be provided regarding the educational program implemented in the target population.	The description of the Gateway programme has been expanded to provide additional contextual information for an international readership.	The following text has been added to the Educational environment and context section: "The Gateway programme includes classroom-based sessions on theoretical concepts, clinical skills, and clinical placements. Taught sessions cover core medical sciences (e.g. anatomy and pharmacology), clinical

		and communication skills (e.g. physical examination and history-taking), social science (e.g. public health and psychology), professionalism, ethics and study skills. The primary focus is facilitating transition from pre-university study to higher education. Following successful completion of the Gateway year, students progress into Yr1, joining the standard medical degree curriculum alongside direct-entry peers."<br><br>We have also added the following sentence for added to the Introduction for additional clarity for an international audience:<br>"In the UK, the primary route into undergraduate medicine is direct entry following completion of secondary education at age 18."
The process of quantitative data collection requires further explanation.	The recruitment strategy for, and administration of, the questionnaire have been described in greater detail.<br><br>We have also included more information in the SRQR and CHERRIES checklists in Supplementary Material 4 and 5.	The following text has been added to the Questionnaire instrument section:<br>"Participation was voluntary and anonymous. A link to the questionnaire was emailed to all Year 2 students (n=242) and advertised using posters, in student newsletters and on social media, and

		mentioned in course news sessions. The survey invitation included details of the study purpose, names of study investigators, details of ethical approval, and the anticipated time for survey completion. Respondents provided electronic informed consent prior to participating. Responses were captured directly in the online survey platform before being downloaded and stored in password-protected cloud storage for analysis. The questionnaire was available for seven weeks at the start of the autumn term.”
The qualitative data analysis process is unclear; the approach, analysis procedure, and steps need to be elaborated.	The qualitative analysis section has been expanded to provide a clearer description of the analytical approach.	The following text has been added to the Questionnaire analysis section:<br>“Free-text responses to questionnaire items were analysed using qualitative content analysis [44]. Responses were read repeatedly to achieve familiarity with the data. Responses were coded according to their meaning, with similar responses grouped into categories developed inductively from the data and categories were refined through iterative review and discussion within the research team. The

		prevalence of categories was examined to identify commonly reported experiences and perceptions, and illustrative quotations were selected to contextualise the quantitative findings."<br><br>The following text has been modified and is now in the new Interview analysis section:<br>"Interview transcripts were read repeatedly to achieve familiarity with the data and analysed using qualitative content analysis [44]. Responses were coded according to their meaning, with similar responses grouped into categories. Categories were initially grouped inductively in line with the questionnaire topics then deductively for new topics [45]. Categories were refined through iterative review and discussion within the research team. Interview participants could comment on topic summaries post-analysis (member checking) [46]. No comments were received."
The method of integrating quantitative and qualitative data is not specified.	We have added a dedicated description explaining when and how quantitative and qualitative findings were integrated and how this	A new section has been added called Data triangulation and the following text added:<br>"Responses to quantitative

	informed interpretation of the results.	questionnaire items were analysed first. Data from both the free-text responses to the questionnaire items and interview transcripts were then analysed together to gain deeper insight into the findings from the quantitative items in the questionnaire.”
Results<br>The study findings need to be rewritten in accordance with the study objectives.	We thank the reviewer for this suggestion. We have reviewed the Results section but believe it appropriately presents the study findings in its current form. None of the other reviewers requested any changes to the Results and, indeed, Review 4 noted “Results are clearly presented”.<br><br>Rather than substantially revise the Results section, we have amended the first paragraph of the Discussion to more explicitly articulate how the reported findings address the study aim and research questions. We believe this change improves clarity without altering the structure of the Results section.	The first paragraph of the Discussion now reads:<br>“To our knowledge, this is the first mixed-methods study to explore the relationship between entry route to medical school and aspects of student experience known to underpin student integration and retention. The study aimed to contrast the perceptions of Gateway-entry and Year 1 direct-entry students on their medical school experience when in Year 2 of the medical degree programme. Regarding our first research question, we found that Gateway-Yr2 had different perceptions of their university experiences compared to direct-entry students in some respects but not others: Gateway-Yr2 respondents were significantly more likely to report feeling disadvantaged, but they were also significantly more likely to feel

		prepared academically than direct-entry respondents, and reported similar levels of non-academic preparedness, sense of belonging and support. Regarding the second research question, Gateway-Yr2 were more likely to undertake paid work but, despite this, most Gateway-Yr2 respondents reported they have the work-life balance they desired. Taken together, we can see that Gateway-Yr2 felt disadvantaged but also felt a sense of belonging; they had to work but also felt they had a reasonable work-life balance. We propose that Gateway may provide opportunities to develop institutional habitus and thus to feel a greater sense of belonging, which is explored below."
**Reviewer 3**		
Clarify the questionnaire development and validation process in slightly more detail.	We thank Reviewer 3 for their helpful comments.<br><br>We have expanded the Questionnaire instrument section to describe questionnaire development, expert review and pilot testing in more detail.	The first paragraph of the Questionnaire instrument section has been amended to: "Questionnaire items were developed following a review of literature relating to belonging, student experiences and support in medical education [for example, 13,14,33–35]. Question items were developed specifically for this study based on the research

		aims. Three colleagues (one final year PhD student and two experienced educators at other medical schools, all with an interest in support of WP students) reviewed the questionnaire items for relevance and clarity. Two current Gateway students reviewed the questionnaire and advertisement text for accessibility, clarity, acceptability and coherence. Minor amendments to phrasing and response options were made in response to their feedback.”
**Reviewer 4**		
Overall evaluation and general comments<br>This is an interesting and well researched study that looks at widening participation in a single medical school in England. It is set in COVID – my assumption is that, if this is like other work, COVID will have magnified issues and reduced contentment in the study population. The study provides insights into the perceptions of students of gateway compared to others in the programme some of whom are also from similar backgrounds to the gateway group and others from more diverse origins. The study suggests gateway eases the route to university/medical programme satisfaction however the authors do	We are grateful for Reviewer 4's thoughtful and encouraging review and appreciate their recognition of the strengths of the study.	

accept they are looking at one group who have two years and another with mostly one year (although including students with previous degree) experience. Learning for me is in the way the gateway programme seems to make the move to the university process better tolerated which is a positive finding, although it is tempered by the fact the study stops at year two when most students will have another 3 years before completion and several years in residency programmes thereafter before reaching career destinations.		
Title and abstract<br>The title reflects the content of the paper and catches the eye. The abstract accurately reflects the content.	We thank the reviewer for this comment.	
Introduction and conceptual framework<br>The introduction sets the scene well. I have no real issues. I do wonder when it became “traditional” for medical students to be from well-heeled, university educated backgrounds? There was a time (in living memory – mine, for example!) when most of the class were students whose parents had been in or had to deal with the aftermath of WW2, rationing, conscription and the like. I’ve never seen a time frame set of the evolution to what we now see. I guess this is me musing but I do wonder about the term “traditional”.	By way of context, Latham et al. (2025) found that only 5% of entrants to medical school in 2021 were from lower socio-economic backgrounds (which represented a doubling since 2012).<br>Cheetham et al. (2025) analysed Labour Force Survey data to estimate the association between socioeconomic background and likelihood of working as a doctor in the UK, and estimate how associations varied over time. They found that “those from professional backgrounds [were] three	We have added both of these references to the end of the first paragraph of the Introduction.

	times more likely to become doctors than those with intermediate backgrounds" and "socioeconomic inequalities were highly stable over time among respondents who turned 18 between the 1960s and the 2000s".	
As regard the conceptual framework, the authors are quite clear in how they set up the study, its structure and theoretical models. I do wonder if when looking at belonging, student number has a role to play? As class sizes mushroom, the ability of staff to provide personal learning opportunities declines, made worse by the apparent attitude on NHS employers in job planning, reducing the time and threatening the ethos of education in the NHS. These may be confounding factors, as may the move in COVID and beyond to more and more online delivery of courses. It might be useful if the authors referenced these other unhelpful pressures in the context of belonging, particularly as they do include a section on wider context which touches on these points.	The Discussion has been amended to acknowledge wider influences on belonging, including smaller class sizes in Gateway, and the impact that larger cohort sizes and an increase in online learning may have on belonging.	We have added this text to the section on Preparedness in the Discussion:<br>"…and the fact that Gateway is generally taught in smaller classes which is known to impact positively on student satisfaction".<br><br>This text has been added to the section on future work:<br>"There are wider influences on belonging which should be borne in mind in any future investigations. This includes larger cohort sizes and online learning becoming more common, which can reduce the availability of personal learning opportunities in both clinical and non-clinical settings."
Methodological rigour<br>Questionnaire instrument<br>I have no issue with the Likert scales. Looking at the	We counted 20 "negative" words (Alarm, Anger, Anxiety, Depressed, Distant,	

question on "words that describe how you feel" (P52, l30) I was struck by the negativity of the words listed. This may well reflect reality but I felt 13 of the 37 were positive whilst the remaining 24 embraced negative perceptions. If someone were to complete this at random, then the likelihood of being negative nearly 3:1 (approx.). I possibly would have just left blank boxes and asked for up to three words positive and three negative to see what we got rather than making the suggestions. I think the authors should comment on this point. (And reference the instrument in the text as appearing in the supplementary material.)	Dread, Empty, Fear, Frustration, Hate, Hostile, Nervous, Panic, Rage, Sad, Shock, Stress, Tension, Terror, Worry) and 17 "positive" words (Calm, Confident, Content, Eager, Enjoyment, Enthusiastic, Excited, Flexible, Happy, Hopeful, Like, Love, Opportunity, Optimistic, Proud, Satisfied, Thankful).<br><br>We do not present any analysis of this question in the manuscript but it remains in the copy of the questionnaire presented in Supplementary Material 2 for accuracy.<br><br>There is an existing reference to the questionnaire in the section titled "Questionnaire instrument".	
Questionnaire analysis<br>I feel the Cronbach's alpha (P12 l46-51) of 0.70 is on the borderline of acceptability – this is stated as the range of "appropriate values" is quoted. A comment on this might be useful to help establish how the authors dealt with this figure given these cut offs have justifications. The use of non-parametric methods is agreed as correct.	We thank the reviewer for highlighting the Chronbach alpha value, and agree that 0.70 is at the lowest boundary of acceptable values. We have therefore re-worded the paragraph as follows for transparency and to expand on its acceptability within the context of exploratory educational research.	The text now reads: "Cronbach alpha, calculated using the Psych package in RStudio [37], deemed the 11 Likert questions presented here to have sufficient internal consistency for analysis (alpha=0.70; appropriate values range between 0.70-0.95) [38]. We acknowledge that this Chronbach alpha value is at the lowest end of the

		appropriate range [39], suggesting that the questionnaire items may measure more than one construct, and results should be interpreted with some caution regarding internal consistency."
Table 1 (P14) mentions the term "care leaver". I assume this means someone who was in care at some point but it might be helpful to state that in brackets after the term to ensure clarity.	The definition for care leaver, along with carer and disability, were given within the demographic questions of the questionnaire. This has been made clearer in the table heading.	This sentence has been added to the heading of Table 1: "Definitions of the terms indicated with * are given within the questionnaire found in Supplementary Material 2".
Interview questions<br>Supplementary material 3 (P59-60) has the question set. A specific question is there about feelings of preparedness for gateway students but there is no easy way to see how this topic was introduced to direct entry students. Would the authors please explain how they ensured that this topic was broached in the interviews. This point does not detract from the information gained in the interviews, but I suspect it does potentially impact on the way the authors compare the groups, even in qualitative work.	This question was asked to Gateway students only to probe how well the Gateway year prepared them for the course. It was not intended as a question about preparedness in general and so was not asked of other students. Preparedness came up in some interviews organically and was probed further where appropriate.	
Results<br>Results are clearly presented but note the point about comparisons above.	We thank Reviewer 4 for this comment.	
Discussion and conclusions<br>The conclusions are clearly stated. The authors do not	We thank Reviewer 4 for this positive comment.	

overstate the significance of the findings rather they are open to the need for further exploration. How feasible it is to tease out factors in a complex and dynamic environment where people are continually developing their professional identity is an interesting point.		
The strengths and limitations (P27 l54-P28 l28) and the summary (P4) seem to me to stress what was done rather than the relevance and possible outcomes. I think a limitation is the timing of the study at year 2 which may be quite early to see if there is long-term benefit. Of course, the longer you wait the more difficult it is to sort out cause and effect that discussion is, I think, a relevant point and it might be helpful to explain why the study was done when it was done. UKMED data research is tending to show a change in residency outcomes to a wider list of specialty career choices in WP groups than the first such study and this might usefully be referenced here.	We have expanded the Limitations section to discuss why this timepoint was selected, acknowledge that the participants we studied were early in their medical training, and note that future longitudinal work is required to determine whether these observations persist.	The following text has been added to the Strengths and limitations section:<br>"We chose to study Yr2 students as a single timepoint of particular interest; it includes transition periods (to university and, for some, from Gateway to Yr1) and is the period when many foundational educational and social experiences are made. Practically, the first cohort of Gateway students at this institution were in Yr2 at the time of the study. Insights from this timepoint allow consideration of early interventions. We acknowledge this is early in medical training and future longitudinal work is required to determine whether these observations persist throughout medical school and into postgraduate training."<br><br>The following text has been added to the Future work section:

		"Recent research has shown that graduates from Gateway programmes face disadvantage when applying for specialty training [63], highlighting the importance of longer-term follow-up".<br><br>We have added this bullet point to the Strengths and Limitations Summary at the start:<br>"Evaluating at Year 2 offers key early insights but limits conclusions about longer-term impacts."
Finally, I am pretty sure COVID will have been influential in this study – but that does not upset the findings, just how the story is told around these, given we mostly felt quite adrift in a sea of uncertainty in the pandemic.	The Strengths and limitations section has been amended to acknowledge the impact of the COVID-19 pandemic on students' experiences and sense of belonging, together with the implications for interpretation of our findings.	The following text has been added to the Strengths and limitations section:<br>"Gateway-Yr2 experienced approximately four months of standard university life before the COVID-19 lockdown restrictions began in England. In comparison, the students who entered Yr1 directly, entered the university when local COVID-19 restrictions prevented in-person teaching taking place (see Supplementary Material 1 for more information). These restrictions are very likely to have magnified issues and reduced contentment in the study population [60,61], particularly in students

		from WP backgrounds [62].”
Clarity length and ethical approval<br>The paper is seriously and quite meticulously written. I cannot fault that. The authors might think about the way the story unfolds and how this lands – although I concede this is a point of style over substance.	We thank Reviewer 4 for this comment.<br><br>We have revised some of the text to improve flow, readability and narrative while maintaining accuracy, bearing in mind the space available.	The name of the local ethics committee has been added.<br><br>Revisions to the text to improve flow are noted in tracked changes.
Ethics – no issue.	We thank Reviewer 4 for this comment.	
EDI<br>Societal structure and function and the inequalities these surface are the essence of the paper.	We thank Reviewer 4 for this comment.	

VERSION 2 - REVIEW

Reviewer 4

Name Johnston, Peter

Affiliation NHS Education for Scotland, North Deanery, Pathology

Date 22-Sep-2026

COI

Many thanks for your work on this paper. I think the current version is good - there are a few minor tiny things the copy editor may well pick up - but I recommend this is published.
