# Supplementary material for: Gateway to belonging? A mixed-methods study exploring the experiences of widening participation students in a UK medical school
Source: BMJ Open. 2026 Oct 15;16(10):e123003. doi: 10.1136/bmjopen-2026-123003 (PMC13691842; doi:10.1136/bmjopen-2026-123003)
Supplement: Supplementary file 2 [file bmjopen-16-10-s001.docx]

# Supplementary Material 1 - further details on timelines relating to the COVID-19 lockdown restrictions in England

This study uses data collected from students in Yr2 of an MBBS course between October and February 2022. Students in this cohort who entered through the Gateway programme began their studies in September 2019. They completed their pre-university qualifications as normal and experienced approximately four months of standard university life before the COVID-19 lockdown restrictions began in England in March 2020. They sat remote open-book exams at the end of Gateway and at the end of Yr1 of the course.

In comparison, students in our study cohort who entered Yr1 directly from school or college, entered the university in September 2020 when England was subject to local COVID-19 restrictions [1] that prevented in-person teaching taking place as in previous academic years. They had also had the end of their pre-university qualifications disrupted (e.g. only limited in-person school teaching was allowed post-March 2020 and A-level exams were cancelled). Students were awarded whichever was higher of either their centre-assessed grades (i.e. the grade their school or college estimated they would most likely have achieved in their examinations) or a moderated version of the centre-assessed grade [2,3]. All students sat remote open-book exams at the end of Yr1.

# Supplementary Material 2 - questionnaire

**Section 1: Experiences whilst at medical school**

**Q: Which programme were you studying when you first became a student at [the medical school]?**

o	Medicine with a Gateway Year

o	Medicine

**Q: Please indicate to what extent you agree or disagree with the following statement using the scale provided.**

**I feel like I belong at medical school.**

o	Disagree

o	Slightly disagree

o	Slightly agree

o	Agree

**If you can, please explain why you gave the answer you did.**

**Q: Please indicate to what extent you agree or disagree with the following statement using the scale provided.**

**I feel prepared academically for Year 2.**

This includes communication skills and working with others.

o	Disagree

o	Slightly disagree

o	Slightly agree

o	Agree

**If you can, please explain why you gave the answer you did.**

**Q: Please indicate to what extent you agree or disagree with the following statement using the scale provided.**

**I feel prepared non-academically for Year 2.**

This can include any non-academic aspect you feel might be relevant e.g. your emotional wellbeing.

o	Disagree

o	Slightly disagree

o	Slightly agree

o	Agree

**If you can, please explain why you gave the answer you did.**

**Q (displayed only if respondent indicated they completed at Gateway Year): Please indicate to what extent you agree or disagree with the following statement using the scale provided.**
 
**I feel the Gateway year prepared me well for Year 1.**

o	Disagree

o	Slightly disagree

o	Slightly agree

o	Agree

**If you can, please explain why you gave the answer you did.**

**Q: Please indicate to what extent you agree or disagree with the following statement using the scale provided.**
 
**I feel my usual workload is manageable.**

o	Disagree

o	Slightly disagree

o	Slightly agree

o	Agree

**If you can, please explain why you gave the answer you did.**

**Q: Please indicate to what extent you agree or disagree with the following statement using the scale provided.**
 
**I feel my workload during the exam period is manageable.**

o	Disagree

o	Slightly disagree

o	Slightly agree

o	Agree

**If you can, please explain why you gave the answer you did.**

**Q: Please indicate to what extent you agree or disagree with the following statement using the scale provided.**
 
**I feel supported by the medical school.**

This can include any staff who you encounter as part of your studies, the Student Support Team and senior leadership in the School.

o	Disagree

o	Slightly disagree

o	Slightly agree

o	Agree

**If you can, please explain why you gave the answer you did.**

**Q: Please indicate to what extent you agree or disagree with the following statement using the scale provided.**
 
**I feel sufficiently confident to ask for any support I need from the medical school.**

This can include any staff who you encounter as part of your studies, the Student Support Team and senior leadership in the School.

o	Disagree

o	Slightly disagree

o	Slightly agree

o	Agree

**Q: Are there any ways the medical school could support you more during your studies? If possible, please give practical examples of useful additional support [the medical school] could provide.**

**Q: Please indicate to what extent you agree or disagree with the following statement using the scale provided.**
 
**I feel that the staff who teach are from a similar background to me.**  

o	Disagree

o	Slightly disagree

o	Slightly agree

o	Agree

**If you can, please explain why you gave the answer you did.**

**Q: Please indicate to what extent you agree or disagree with the following statement using the scale provided.**
 
**I *want to* take part in extra-curricular activities.**  

This can include sport, student societies, voluntary roles, etc.

o	Disagree

o	Slightly disagree

o	Slightly agree

o	Agree

**If you can, please explain why you gave the answer you did.**

**Q: Please indicate to what extent you agree or disagree with the following statement using the scale provided.**
 
**I *can* take part in extra-curricular activities to the extent that I would like to.**

This can include sport, student societies, voluntary roles, etc.

o	Disagree

o	Slightly disagree

o	Slightly agree

o	Agree

**If you can, please explain why you gave the answer you did.**

**Q: Do you undertake paid employment?**

o	Yes - approximately the same hours every week

o	Yes - hours worked fluctuates each week

o	Yes - on vacation periods only

o	Yes - other (please use the text box to provide further information if you would like to)

o	No

o	Prefer not to say

**Q (displayed only if respondent indicated they undertook paid employment): Please indicate to what extent you agree or disagree with the following statement using the scale provided.**
 
**It is necessary for me to work in order to support myself financially.**

o	Disagree

o	Slightly disagree

o	Slightly agree

o	Agree

**If you can, please explain why you gave the answer you did.**

**Q (displayed only if respondent indicated they undertook paid employment): Please indicate to what extent you agree or disagree with the following statement using the scale provided.**

**My paid employment impacts on my studies and university experience.**

o	Disagree

o	Slightly disagree

o	Slightly agree

o	Agree

**If you can, please explain why you gave the answer you did.**

**Q: Are you considering studying for an intercalated degree?**

An intercalated degree is an opportunity for medical students to take a year of absence from their medical training to undertake a further degree qualification.

o	Yes

o	No

o	Undecided

o	Have not thought about it

o	Prefer not to say

**If you can, please explain why you gave the answer you did.**

**Q: Please indicate to what extent you agree or disagree with the following statement using the scale provided.**
 
**I feel that I have the work-life balance I would like.**

o	Disagree

o	Slightly disagree

o	Slightly agree

o	Agree

**If you can, please explain why you gave the answer you did.**

**Q: Has your relationship with your family changed since you started at medical school?**

o	No

o	Yes - better

o	Yes - worse

o	Yes - mixed

o	Not sure

o	Other

o	Prefer not to say

**If you can, please explain why you gave the answer you did.**

**Q: Has your relationship with the friends you had before medical school changed since you started at medical school?**

o	No

o	Yes - better

o	Yes - worse

o	Yes - mixed

o	Not sure

o	Other

o	Prefer not to say

**If you can, please explain why you gave the answer you did.**

**Q: Have you ever felt that your background has disadvantaged you in any way during your time at medical school?**

o	No

o	Yes - occasionally

o	Yes - many times

o	Not sure

o	Other

o	Prefer not to say

**If you can, please explain why you gave the answer you did.**

**Q: Which of these words best describes how you feel about your current experience of medical school? Select all that apply.**

▢	Alarm

▢	Anger

▢	Anxiety

▢	Calm

▢	Confident

▢	Content

▢	Depressed

▢	Distant

▢	Dread

▢	Eager

▢	Empty

▢	Enjoyment

▢	Enthusiastic

▢	Excited

▢	Fear

▢	Flexible

▢	Frustration

▢	Happy

▢	Hate

▢	Hopeful

▢	Hostile

▢	Like

▢	Love

▢	Nervous

▢	Opportunity

▢	Optimistic

▢	Panic

▢	Proud

▢	Rage

▢	Sad

▢	Satisfied

▢	Shock

▢	Stress

▢	Tension

▢	Terror

▢	Thankful

▢	Worry

▢	Other (please write additional words in the text box if you would like to)

**Section 2: Experiences prior to medical school**

**Q: Please indicate to what extent you agree or disagree with the following statement using the scale provided.**
 
**My family supported me when I was applying to medical school.**

This can include any type of support: moral, emotional, financial, logistical, etc.

o	Disagree

o	Slightly disagree

o	Slightly agree

o	Agree

**If you can, please explain why you gave the answer you did.**

**Q: Please indicate to what extent you agree or disagree with the following statement using the scale provided.**
 
**My school, college or university supported me when I was applying to medical school.**

This can include any type of support: moral, emotional, financial, logistical, etc.

o	Disagree

o	Slightly disagree

o	Slightly agree

o	Agree

**If you can, please explain why you gave the answer you did.**

**Q: Did you complete a recognised widening participation/widening access scheme prior to entering medical school?** 

For example, Pathways to Medicine, [the medical school] Year 12 Summer School, widening participation/widening access schemes run by other medical schools.

o	Yes

o	No

o	Not sure

o	Other (please use the text box to provide further information if you would like to)

o	Prefer not to say

**Q (displayed only if respondent indicated they complete a recognised widening participation/widening access): How useful was this to you?**

Leave blank if you prefer not to say.

**Section 3: About you**

**Q: Did you live in the UK immediately prior to starting medical school?**

o	Yes

o	No

o	Prefer not to say

**Q (displayed only if respondent indicated they lived in the UK immediately prior to starting medical school):** **What was the postcode of the place you lived immediately before you started medical school? This may be the same as your current postcode.**

Please only include the first part of the postcode.

Leave blank if you prefer not to say.

**Q: What type of school or college did you attend to study for your pre-university qualifications?** 

This is usually a national examination taken at the age of 18 and could include A-levels, Scottish Highers, International Baccalaureate, Irish Leaving Certificate, BTECs, T-levels or equivalent.

o	Non-selective

o	Selective

o	Fee-paying school (including if you or your family did not pay the fees yourself)

o	State/government-funded school

o	Home-schooled

o	Other (please use the text box to provide further information if you would like to)

o	Prefer not to say

**Q (displayed only if respondent indicated they lived in the UK immediately prior to starting medical school): Did you receive free school meals?**

o	Yes

o	No

o	Not sure

o	Other (please use the text box to provide further information if you would like to)

o	Prefer not to say

**Q (displayed only if respondent indicated they received free school meals): For how many years were you in receipt of free school meals?** 

Leave blank if you prefer not to say.

**Q (displayed only if respondent indicated they lived in the UK immediately prior to starting medical school): Were you in receipt of the Educational Maintenance Allowance or a 16 to 19 Bursary?**

o	Yes

o	No

o	Not sure

o	Prefer not to say

**Q: What is the highest level of education your parents or guardians completed?** 

If your parents or guardians have different educational achievements, choose the higher level.

o	No formal qualifications

o	GCSEs/O levels/high school qualification at 16 years old

o	A levels/high school qualification at 18 years old/Access course

o	Vocational qualification e.g. BTEC, GNVQ

o	Undergraduate degree e.g. BSc, BA, MBChB, DBS

o	Masters degree e.g. MSc, MA, MBA

o	Higher degree/doctorate e.g. PhD, MD, DPhil

o	Other (please use the text box to provide further information if you would like to)

o	Prefer not to say

**Q: Do any members of your immediate family that you are in regular contact with work as doctors?**

Select all that are applicable.

If they worked as a doctor in the past but no longer do, count this as “yes”.

If your grandparents, aunt, uncle, sibling, etc., were your guardian, select the guardian option if it is applicable rather than the grandparent/aunt/uncle/sibling option.

▢	Yes - parents/guardians

▢	Yes - grandparents/aunt/uncle/sibling

▢	Yes - cousin/husband/wife/civil partner

▢	No

▢	Other (please use the text box to provide further information if you would like to)

▢	Prefer not to say

**Q: Do any members of your immediate family that you are in regular contact with work as other health care professionals?**

Select all that are applicable.

If they worked as a health care professional in the past but no longer do, count this as “yes”.
 
If your grandparents, aunt, uncle, etc., were your guardian, select the guardian option if it is applicable rather than the grandparent/aunt/uncle option.

▢	Yes - parents/guardians

▢	Yes - grandparents/aunt/uncle/sibling

▢	Yes - cousin/husband/wife/civil partner

▢	No

▢	Other (please use the text box to provide further information if you would like to)

▢	Prefer not to say

**Q (displayed only if respondent indicated they had parents/guardians, grandparents/aunt/uncle/sibling or cousin/husband/wife/civil partner who worked as a healthcare professional): What was/is their profession(s)?**

Leave blank if you prefer not to say.

**Q: Are you currently, or have you previously been, in receipt of a bursary?**

o	Yes - UCAT bursary

o	Yes - other bursary (please use the text box to provide further information if you would like to)

o	No

o	Not sure

o	Prefer not to say

**Q: Are you a carer?** 

The NHS defines carer as anyone who “looks after a family member, partner or friend who needs help because of their illness, frailty, disability, a mental health problem or an addiction and cannot cope without their support”. This support is unpaid.

o	Yes

o	No

o	Not currently but was in the past

o	Other (please use the text box to provide further information if you would like to)

o	Prefer not to say

**Q: How would you describe your ethnic background?**

o	Asian (including British Asian, Bangladeshi, Indian and Pakistani)

o	Arab

o	Black (including Black British, Black African and Black Caribbean)

o	Chinese

o	Gypsy, Roma, Traveller, Showman or Boater

o	Mixed/multiple (including White and Asian, White and Black African, White and Black Caribbean)

o	White (including British, European and Irish)

o	Other (please use the text box to provide further information if you would like to)

o	Prefer not to say

**Q: Do you have a disability?** 

This includes mental or physical disabilities or specific learning differences e.g. dyslexia.  The Equality Act 2010 defines a disabled person as “someone who has a mental or physical impairment that has a substantial and long-term adverse effect on the person’s ability to carry out normal day-to-day activities.”

o	Yes

o	No

o	Not sure

o	Other (please use the text box to provide further information if you would like to)

o	Prefer not to say

**Q: Are you a care leaver?** 

The Children (Leaving Care) Act 2000 defines a care leaver as someone who has been in the care of the Local Authority i.e. there was a period of time when you were cared for by the Local Authority and not your parents or close family.

o	Yes

o	No

o	Not sure

o	Other (please use the text box to provide further information if you would like to)

o	Prefer not to say

**Q: How would you describe your gender?**

o	Female/woman

o	Male/man

o	Non-binary

o	Other (please use the text box to provide further information if you would like to)

o	Prefer not to say

**Q: Did you complete an undergraduate degree prior to entering medical school?**

o	Yes

o	No

o	Other (please use the text box to provide further information if you would like to)

o	Prefer not to say

**Q (displayed only if respondent indicated they complete an undergraduate degree prior to entering medical school): Which subject(s) did you study?**

Leave blank if you prefer not to say.

**Q: How old are you?**

o	18-21 years

o	22-25 years

o	26+ years

o	Prefer not to say

**Q: Do you speak English as an additional language?**

o	No

o	Yes - second language

o	Yes - third+ language

o	Other (please use the text box to provide further information if you would like to)

o	Prefer not to say

**Q:** **If you have any final comments or responses, please write them below.**

# Supplementary Material 3 - semi-structured interview questions

## Course/academic skills

Open question to start: **How is the course going?**

### If lost/gained experiences due to COVID-19 comes up:

- What do you feel you **lack most or have gained** as a result of the pandemic? Academic and non-academic.
- Has **COVID-19 exacerbated the pressure to over-commit/catch-up** now? e.g. socialising, making friends, work opportunities. In what ways?

### If academic skills development comes up:

- Which **skills do you feel confident in**/which would you have liked help in developing e.g. note-taking, revising, time management? How would you **like that delivered**?

## Gateway

- **For Gateway students** - how did Gateway help prepare for Year 1 and Year 2?

## Work

- **Do you work**? Do you need to work to support yourself? How does this affect your studies/extra-curricular activities?
- If you don’t work, what do you think of students who do?

## Background

- Have you ever felt that your **background has disadvantaged you** in any way during your time at medical school? Please explain.
- Have you felt like you needed to **change your behaviour** in any way whilst at medical school? Please explain.
- Do you **feel like you are part of an under-represented group** at medical school?
- If yes, how does being part of an under-represented group at medical school affect your **relationship with patients, other students, staff**?
- Do you feel the **staff represent/understand where you are coming from**?
- How does being at medical school affect your **relationship with your family/the friends** you had before medical school? Do you feel supported by your family?
- Students from under-represented backgrounds are referred to by a number of terms. **What terms do you know and prefer**? Examples from the literature include “widening participation”, “first-in-family”, “working class”, “non-traditional”, “under-represented minority”, “under-represented group”. How do you feel about these terms? Do you identify with any of these? For Gateway students - this medical school uses the term “Gateway Year” to describe what other medical schools call a “Foundation Year” or “Year 0”. Do you prefer any of these terms?

## Support from the medical school

- Is there **any support** you would like the medical school to have in place to help students from under-represented backgrounds?
- Could the medical school do anything more to support applying to medical school?

## Future

- How do you view your **future journey** to becoming a doctor?

Is there anything you would like to add that I haven’t already asked you about?

# Supplementary Material 4 - SRQR checklist

| **Topic** | **Reporting Item** | **Location (or reason for not**  **reporting)** |
| --- | --- | --- |
| **Title and abstract** | | |
| Title | Concise description of the nature and topic of the study Identifying the study as qualitative or indicating the approach (e.g., ethnography, grounded theory) or data collection methods (e.g., interview, focus group) is recommended | Title |
| Abstract | Summary of key elements of the study using the abstract format of the intended publication; typically includes background, purpose, methods, results, and conclusions | Abstract |
| **Introduction** | | |
| Problem formulation | Description and significance of the problem/phenomenon studied; review of relevant theory and empirical work; problem statement | Introduction |
| Purpose or research question | Purpose of the study and specific objectives or questions | Introduction |
| **Methods** | | |
| Qualitative approach and research paradigm | Qualitative approach (e.g., ethnography, grounded theory, case study, phenomenology, narrative research) and guiding theory if appropriate; identifying the research paradigm (e.g., postpositivist, constructivist/ interpretivist) is also recommended; rationale | Introduction and Methods |
| Researcher characteristics and reflexivity | Researchers’ characteristics that may influence the research, including personal attributes, qualifications/experience, relationship with participants, assumptions, and/or presuppositions; potential or actual interaction between researchers’ characteristics and the research questions, approach, methods, results, and/or transferability | Methods |
| Context | Setting/site and salient contextual factors; rationale | Methods |
| Sampling strategy | How and why research participants, documents, or events were selected; criteria for deciding when no further sampling was necessary (e.g., sampling saturation); rationale | Methods |
| Ethical issues pertaining to human subjects | Documentation of approval by an appropriate ethics review board and participant consent, or explanation for lack thereof; other confidentiality and data security issues | Methods |
| Data collection methods | Types of data collected; details of data collection procedures including (as appropriate) start and stop dates of data collection and analysis, iterative process, triangulation of sources/methods, and modification of procedures in response to evolving study findings; rationale | Methods |
| Data collection instruments and technologies | Description of instruments (e.g., interview guides, questionnaires) and devices (e.g., audio recorders) used for data collection; if/how the instrument(s) changed over the course of the study | Methods |
| Units of study | Number and relevant characteristics of participants, documents, or events included in the study; level of participation (could be reported in results) | Results |
| Data processing | Methods for processing data prior to and during analysis, including transcription, data entry, data management and security, verification of data integrity, data coding, and anonymization/de-identification of excerpts | Methods |
| Data analysis | Process by which inferences, themes, etc., were identified and developed, including the researchers involved in data analysis; usually references a specific paradigm or approach; rationale | Methods and Results |
| Techniques to enhance trustworthiness | Techniques to enhance trustworthiness and credibility of data analysis (e.g., member checking, audit trail, triangulation); rationale | Methods |
| **Results/findings** | | |
| Synthesis and interpretation | Main findings (e.g., interpretations, inferences, and themes); might include development of a theory or model, or integration with prior research or theory | Discussion |
| Links to empirical data | Evidence (e.g., quotes, field notes, text excerpts, photographs) to substantiate analytic findings | Discussion |
| **Discussion** | | |
| Integration with prior work, implications, transferability, and contribution(s) to the field | Short summary of main findings; explanation of how findings and conclusions connect to, support, elaborate on, or challenge conclusions of earlier scholarship; discussion of scope of application/generalizability; identification of unique contribution(s) to scholarship in a discipline or field | Discussion |
| Limitations | Trustworthiness and limitations of findings | Discussion |
| **Other** | | |
| Conflicts of interest | Potential sources of influence or perceived influence on study conduct and conclusions; how these were managed | End of manuscript |
| Funding | Sources of funding and other support; role of funders in data collection, interpretation, and reporting | End of manuscript |

# Supplementary Material 5 - CHERRIES checklist

| **Checklist Item** | **Explanation** | **Description** | **Location** |
| --- | --- | --- | --- |
| **Design** | | | |
| Describe survey design | Describe target population, sample frame. Is the sample a convenience sample? (In “open” surveys this is most likely.) | The target population was Year 2 medical students at a UK medical school.  The questionnaire was promoted internally to all Yr2 students for seven weeks at the start of the autumn term.  The sample is not a convenience sample. | Methods |
| **Institutional Review Board (IRB) approval** | | | |
| IRB approval | Mention whether the study has been approved by an IRB. | Ethical approval was obtained from Hull York Medical School Ethics Committee (reference number 21 48). | Methods |
| Informed consent | Describe the informed consent process. Where were the participants told the length of time of the survey, which data were stored and where and for how long, who the investigator was, and the purpose of the study? | The information at the top of the questionnaire explained the expected time needed to complete the questionnaire, who the investigator was, and the purpose of the study. Participants were also told that all responses were collected anonymously and that participation was voluntary. Informed consent was given after reading information at the top of the questionnaire. | Methods |
| Data protection | If any personal information was collected or stored, describe what mechanisms were used to protect unauthorized access. | Data were held in Microsoft Teams/OneDrive which was password-protected. Only the researchers involved with the study had access to raw data. Use of email was kept to a minimum and only contained anonymised data. | Methods |
| **Development and pretesting** | | | |
| Development and testing | State how the survey was developed, including whether the usability and technical functionality of the electronic questionnaire had been tested before fielding the questionnaire. | Ideas from the literature contributed to the design of the questionnaire. Feedback on the questions was obtained from three colleagues and two students. Their specific comments were incorporated into the final question design. A draft questionnaire was tested for technical functionality by the research team. | Methods |
| **Recruitment process and description of the sample having access to the questionnaire** | | | |
| Open survey versus closed survey | An “open survey” is a survey open for each visitor of a site, while a closed survey is only open to a sample which the investigator knows (password-protected survey). | A closed questionnaire was used, only open to a specific population. | Methods |
| Contact mode | Indicate whether or not the initial contact with the potential participants was made on the Internet. (Investigators may also send out questionnaires by mail and allow for Web-based data entry.) | Participants were contacted by virtue of being part of a student mailing list (e.g. by email or student newsletter). Posters and social media posts containing QR codes/links were also available to potential participants. | Methods |
| Advertising the survey | How/where was the survey announced or advertised? Some examples are offline media (newspapers), or online (mailing lists – If yes, which ones?) or banner ads (Where were these banner ads posted and what did they look like?). It is important to know the wording of the announcement as it will heavily influence who chooses to participate. Ideally the survey announcement should be published as an appendix. | Electronic mailing lists were used to contact potential participants, as well as QR codes/links on posters and social media. | Methods |
| **Survey administration** | | | |
| Web/E-mail | State the type of e-survey (e.g., one posted on a Web site, or one sent out through e-mail). If it is an e-mail survey, were the responses entered manually into a database, or was there an automatic method for capturing responses? | The questionnaire was electronic and hosted on Qualtrics. Responses were captured directly in the online survey platform before being downloaded and stored in OneDrive/Teams for analysis. Responses were multiple choice and open text. | Methods |
| Context | Describe the Web site (for mailing list/newsgroup) in which the survey was posted. What is the Web site about, who is visiting it, what are visitors normally looking for? Discuss to what degree the content of the Web site could pre-select the sample or influence the results. For example, a survey about vaccination on an anti-immunization Web site will have different results from a Web survey conducted on a government Web site | Not applicable. | Not applicable |
| Mandatory/voluntary | Was it a mandatory survey to be filled in by every visitor who wanted to enter the Web site, or was it a voluntary survey? | Completion of questionnaire was voluntary. | Methods |
| Incentives | Were any incentives offered (e.g., monetary, prizes, or non-monetary incentives such as an offer to provide the survey results)? | No. | Methods |
| Time/Date | In what timeframe were the data collected? | The questionnaire was promoted internally to all Year 2 students for seven weeks at the start of the autumn term. | Methods |
| Randomisation of items or questionnaires | To prevent biases items can be randomised or alternated. | No randomisation of items was used. | Methods |
| Adaptive questioning | Use adaptive questioning (certain items, or only conditionally displayed based on responses to other items) to reduce number and complexity of the questions. | Adaptive questioning (branching) was used where appropriate. Relevant survey items were displayed based on previous responses (e.g. only those who had completed a Gateway Year were asked how well the Gateway Year prepared them for Year 1). | Methods |
| Number of Items | What was the number of questionnaire items per page? The number of items is an important factor for the completion rate. | A maximum of two items were displayed on a questionnaire page. | Methods |
| Number of screens (pages) | Over how many pages was the questionnaire distributed? The number of items is an important factor for the completion rate. | The full questionnaire was distributed over up to 53 pages. Not all questions were visible to all respondents based on adaptive questioning. | Methods |
| Completeness check | It is technically possible to do consistency or completeness checks before the questionnaire is submitted. Was this done, and if “yes”, how (usually JAVAScript)? An alternative is to check for completeness after the questionnaire has been submitted (and highlight mandatory items). If this has been done, it should be reported. All items should provide a non-response option such as “not applicable” or “rather not say”, and selection of one response option should be enforced. | The questionnaire contained mandatory and voluntary questions. Many questions were followed by a question asking “If you can, please explain why you gave the answer you did” which was voluntary. Most items, except consent items and those items required for adaptive questioning, included a “Prefer not to answer” option. | Methods |
| Review step | State whether respondents were able to review and change their answers (e.g., through a Back button or a Review step which displays a summary of the responses and asks the respondents if they are correct). | Respondents were able to move back to previous questions and change their answers. They were also able to come back within one week and complete their answers later. | Methods |
| **Response rates** | | | |
| Unique site visitor | If you provide view rates or participation rates, you need to define how you determined a unique visitor. There are different techniques available, based on IP addresses or cookies or both. | Not applicable. | Not applicable |
| View rate (Ratio of unique survey visitors/unique site visitors) | Requires counting unique visitors to the first page of the survey, divided by the number of unique site visitors (not page views!). It is not unusual to have view rates of less than 0.1 % if the survey is voluntary. | We did not count the number of unique visitors to the first page of the survey. | Not applicable |
| Participation rate (Ratio of unique visitors who agreed to participate/unique first survey page visitors) | Count the unique number of people who filled in the first survey page (or agreed to participate, for example by checking a checkbox), divided by visitors who visit the first page of the survey (or the informed consents page, if present). This can also be called “recruitment” rate. | Not applicable. | Not applicable |
| Completion rate (Ratio of users who finished the survey/users who agreed to participate) | The number of people submitting the last questionnaire page, divided by the number of people who agreed to participate (or submitted the first survey page). This is only relevant if there is a separate “informed consent” page or if the survey goes over several pages. This is a measure for attrition. Note that “completion” can involve leaving questionnaire items blank. This is not a measure for how completely questionnaires were filled in. (If you need a measure for this, use the word “completeness rate”.) | 107 agreed to participate and 69 submitted the last questionnaire page, which gives a completion rate of 64%. | Methods |
| **Preventing multiple entries from the same individual** | | | |
| Cookies used | Indicate whether cookies were used to assign a unique user identifier to each client computer. If so, mention the page on which the cookie was set and read, and how long the cookie was valid. Were duplicate entries avoided by preventing users access to the survey twice; or were duplicate database entries having the same user ID eliminated before analysis? In the latter case, which entries were kept for analysis (e.g., the first entry or the most recent)? | Cookies were not used. | Not applicable |
| IP check | Indicate whether the IP address of the client computer was used to identify potential duplicate entries from the same user. If so, mention the period of time for which no two entries from the same IP address were allowed (e.g., 24 hours). Were duplicate entries avoided by preventing users with the same IP address access to the survey twice; or were duplicate database entries having the same IP address within a given period of time eliminated before analysis? If the latter, which entries were kept for analysis (e.g., the first entry or the most recent)? | The online survey platform was set to not collect IP addresses. | Not applicable |
| Log file analysis | Indicate whether other techniques to analyze the log file for identification of multiple entries were used. If so, please describe. | Not used. | Not applicable |
| Registration | In “closed” (non-open) surveys, users need to login first and it is easier to prevent duplicate entries from the same user. Describe how this was done. For example, was the survey never displayed a second time once the user had filled it in, or was the username stored together with the survey results and later eliminated? If the latter, which entries were kept for analysis (e.g., the first entry or the most recent)? | Registration was not used. | Not applicable |
| **Analysis** | | | |
| Handling of incomplete questionnaires | Were only completed questionnaires analyzed? Were questionnaires which terminated early (where, for example, users did not go through all questionnaire pages) also analyzed? | Incomplete questionnaires were recorded but were not included in analysis. | Methods |
| Questionnaires submitted with an atypical timestamp | Some investigators may measure the time people needed to fill in a questionnaire and exclude questionnaires that were submitted too soon. Specify the timeframe that was used as a cut-off point, and describe how this point was determined. | No respondents were removed from the survey for completing the questionnaire too quickly. | Methods |
| Statistical correction | Indicate whether any methods such as weighting of items or propensity scores have been used to adjust for the non-representative sample; if so, please describe the methods. | Not used. | Not applicable |

# Supplementary Material 6 - extra-curricular activities

Nearly all students (66/69=96%) agreed that they wanted to take part in extra-curricular activities. Across all respondents there was a significant difference in the level of agreement between the questions “I *want* to take part in extra-curricular activities” and “I *can* take part in extra-curricular activities to the extent that I would like to” (U=3541.5, p<0.001, with 44/69 (64%) agreeing to the latter statement). There were no significant differences between Gateway-Yr2 and direct-entry for either question (Figure S.4.1). Free-text comments and interviews explained extra-curricular activities provide a way to de-stress, meet new people with common interests, improve wellbeing, and be part of a community.

One interview participant mentioned the value of medics’ sports teams and societies to meet medical students in other years and exchange useful information:

*We talked about [the upcoming clinical examinations] a couple of weeks ago … saying that if you …. don’t get stuff sorted now … it becomes harder … later on.* (Interviewee E)

*For careers advice I think [student societies] are quite good for that…; I’m doing… [a] trauma orthopaedics one on Tuesday… about getting onto core surgical training.* (Interviewee E)

Reasons given for not being able to take part in extra-curricular activities included a lack of time but also (from some) an acknowledgement that it would be impossible to do everything:

*There's no time.* (direct-entry respondent, WP)

Extra-curricular activities can be beneficial for mental and physical health [4–7], belonging [8], student success [9,10] and also integration into a medical profession [11–13]. Participants are significantly more likely to agree that they *want* to take part in extra-curricular activities than *can*. The main reason was lack of time.

WP students may be less likely to engage with extra-curricular activities as they might have caring responsibilities, paid work or other obligations [14]. Medic clubs and societies are a good way to meet medical students in other years and is an opportunity to pass on tacit information [15]; students who have to work (or have other obligations) miss out on this part of the hidden curriculum. WP students may have been the only person in their school applying to medicine, not know any doctors personally, and need to work to support themselves, thus missing out on networking opportunities [16,17].

**Figure S.4.1:** **Student perceptions of work-life balance at medical school by entry pathway.** For each question positive responses (agree) are on the right, negative responses (disagree) on the left. Sample sizes: direct-entry n=54, Gateway n=15. Results of Mann-Whitney U test indicated.

# References in Supplementary Material

1	Brown J, Kirk-Wade E. Coronavirus: A history of English lockdown laws [online]. 2021. https://commonslibrary.parliament.uk/research-briefings/cbp-9068/ (accessed 30 January 2025)

2	Ofqal. Statement from Roger Taylor, Chair, Ofqual [online]. 2020. https://www.gov.uk/government/news/statement-from-roger-taylor-chair-ofqual (accessed 30 January 2025)

3	Ofqal. GCSE, AS and A level: Autumn and November 2020 exam series [online]. 2021. https://www.gov.uk/government/publications/gcse-as-and-a-level-autumn-and-november-2020-exam-series (accessed 30 January 2025)

4	Finnerty R, Marshall SA, Imbault C, *et al.* Extra-curricular activities and well-being: Results from a survey of undergraduate university students during COVID-19 lockdown restrictions. *Front Psychol*. 2021;12:647402. doi: 10.3389/fpsyg.2021.647402

5	Herbert C. Enhancing mental health, well-being and active lifestyles of university students by means of physical activity and exercise research programs. *Front Public Health*. 2022;10:849093. doi: 10.3389/fpubh.2022.849093

6	Manley AE, Biddle L, Savović J, *et al.* The positive and negative consequences of stress and its relationship with coping in medical students: A qualitative study. *Med Teach*. 2024;1–6. doi: 10.1080/0142159X.2024.2333799

7	Rhodes RE, Janssen I, Bredin SSD, *et al.* Physical activity: Health impact, prevalence, correlates and interventions. *Psychol Health*. 2017;32:942–75. doi: 10.1080/08870446.2017.1325486

8	Knifsend CA. Intensity of activity involvement and psychosocial well-being among students. *Act Learn High Educ*. 2020;21:116–27. doi: 10.1177/1469787418760324

9	King AE, McQuarrie FAE, Brigham SM. Exploring the relationship between student success and participation in extracurricular activities. *Sch J Leis Stud Recreat Educ*. 2021;36:42–58. doi: 10.1080/1937156X.2020.1760751

10	Ribeiro N, Malafaia C, Neves T, *et al.* The impact of extracurricular activities on university students’ academic success and employability. *Eur J High Educ*. 2024;14:389–409. doi: 10.1080/21568235.2023.2202874

11	Kim S, Jeong H, Cho H, *et al.* Extracurricular activities in medical education: an integrative literature review. *BMC Med Educ*. 2023;23:278. doi: 10.1186/s12909-023-04245-w

12	Raja U, Amin MSA, Soualhi A. Twelve tips to maximise medical student exposure during surgical placements. *Med Teach*. 2021;43:637–41. doi: 10.1080/0142159X.2020.1831465

13	Vasilica A-M, Kumar NS. Twelve tips for successfully getting involved in research as a medical student. *Med Teach*. 2022;44:962–6. doi: 10.1080/0142159X.2021.1915469

14	Stuart M, Lido C, Morgan J, *et al.* The impact of engagement with extracurricular activities on the student experience and graduate outcomes for widening participation populations. *Act Learn High Educ*. 2011;12:203–15. doi: 10.1177/1469787411415081

15	Hordósy R, Clark T. Beyond the compulsory: a critical exploration of the experiences of extracurricular activity and employability in a northern red brick university. *Res Post-Compuls Educ*. 2018;23:414–35. doi: 10.1080/13596748.2018.1490094

16	Brosnan C, Southgate E, Outram S, *et al.* Experiences of medical students who are first in family to attend university. *Med Educ*. 2016;50:842–51. doi: 10.1111/medu.12995

17	Bassett AM, Brosnan C, Southgate E, *et al.* The experiences of medical students from First-in-Family (FiF) university backgrounds: a Bourdieusian perspective from one English medical school. *Res Post-Compuls Educ*. 2019;24:331–55. doi: 10.1080/13596748.2018.1526909
